# Antiadhesive and Antibacterial Coatings for Short‐Term Titanium Implants

**DOI:** 10.1002/marc.202400989

**Published:** 2025-03-08

**Authors:** Fiona Wiesner, Karl G. M. Schönewald, Kira Vogel, Niklas Jung, Barbara Schwierz, Maren Kipping, Aliia Ibragimova, Joshua Schumacher, Christian Pritzel, Clinton R. V. Thiagarajan, Ulrike Ritz, Ulrich Jonas

**Affiliations:** ^1^ University of Siegen, Department of Biology and Chemistry Macromolecular Chemistry Adolf‐Reichwein‐Straße 2 57076 Siegen Germany; ^2^ University Medical Center Mainz Department of Orthopedics and Trauma Surgery Langenbeckstraße 1 55131 Mainz Germany; ^3^ University of Siegen, Department of Biology and Chemistry Physical Chemistry I Adolf‐Reichwein‐Straße 2 57076 Siegen Germany; ^4^ University of Siegen, Department of Biology and Chemistry Chemistry of Building Materials Paul‐Bonatz‐Straße 9‐11 57076 Siegen Germany

**Keywords:** antiadhesive and antibacterial hydrogel coatings, antifouling, fibroblasts, photocrosslinkable poly(acrylamides), short‐term titanium implants, tenocytes

## Abstract

This study presents a novel approach for the development of antifouling and antibacterial hydrogel coatings for short‐term titanium implants to treat bone defects. Such implants provide temporary stabilization during bone healing and are intended to be explanted within a period of 12 months. The novel surface modification prevents complications during implant removal, like injury to tissue, nerves, or tendons due to adhesion to the untreated titanium surface. The coatings combine acrylamide‐based hydrogels with photocrosslinkers possessing intrinsic antibacterial properties and anchor groups designed for titanium substrates. Comprehensive in vitro evaluations are conducted to assess the biocompatibility and efficacy of these coatings. The results demonstrate that the water‐swellable polymer networks effectively prevent cell and tissue adhesion by their antifouling characteristics without inducing cytotoxicity. Importantly, these coatings also exhibit an intrinsic and non‐leaching antibacterial effect covalently incorporated into the molecular framework, which addresses the limitations of current implant coating technologies that often rely on the incorporation of antibiotics or bactericidal agents. As the experimental data conclusively verify the effectiveness of the coatings in inhibiting cell adhesion and bacterial colonization, this technology shows great potential to significantly advance the field of short‐term titanium implants.

## Graphical abstract

An innovative concept is presented for antifouling and antibacterial hydrogel coatings for short‐term titanium implants that provide temporary stabilization during the healing of bone defects with intended explantation within one year. The acrylamide‐based hydrogel modification with intrinsic antibacterial properties targets complications during implant removal, like injury to tissue, nerves, or tendons due to adhesion to the untreated titanium surface.

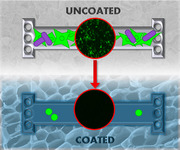

## 1 Introduction

Fractures are a worldwide occurring problem with a variety of different treatment possibilities depending on severity, localization, and risk factors. The analysis of the Global Burden of Diseases, Injuries, and Ris Factors Study (GBD) reported with 178 million new fractures, the large burden of fractures worldwide in the year 2019. Since 1990 the absolute number increased by 33.4% demonstrating the elevation of fractures in the elder society.^[^ ^1^ ^]^ In Germany, the observed increase was by 14% (from 2009 to 2019).^[^ ^2^ ^]^

Depending on the type of fracture screws and/ or plates are surgically inserted to join and fix the bone fragments in an anatomically correct position, known as osteosynthesis. The duration of the metal implants depends on the type and localization of the fracture. In the case of hip or knee implants, it is desirable that the implant lasts as long as possible and integrates completely into the tissue.^[^ ^3^ ^]^ Other implants have to be removed after some time; in this case, it is desirable, that the implant does not connect with the surrounding tissue to facilitate its removal without any tissue damage.^[^ ^4^ ^]^ Implant removal currently accounts for approx. 30% of all elective operations in trauma surgery/orthopedics. If the fracture heals without complications, removal at the distal radius is recommended after 3–6 months, particularly after 9–12 months and diaphyseally after 12–24 months.^[^ ^5^, ^6^ ^]^

Adhesion is a frequent complication when removing osteosynthesis material. Adhesions describe an abnormal hyperplasia of tissue that can take on different dimensions. It can mean the formation of a thin fibrous layer, but also thick scar tissue. This is often associated with chronic pain, dysfunction, and reduced quality of life for the patient.^[^ ^7^ ^]^ After insertion of the implants, fibrous scar tissue forms as part of the defect healing process, and connective tissue adhesions to the implant occur. Tendons can also be affected, which can lead to permanent reduced mobility of the joint and periarthritis. This often leads to adhesion of the tendon to the material, resulting in an increased risk of tenosynovitis and tendon rupture. The most common and most serious complication is tenogenic stiffening of the joint. If material is subsequently removed, tenolysis is often indicated, in which the tendon is detached from the surrounding tissue, although the prognosis is poor and there is a risk of irreversible loss of function. The only safe strategy would be to prevent tendon adhesion.^[^ ^4^, ^8^ ^]^ Another serious complication is infection of the osteosynthesis material. The bacterium Staphylococcus aureus is particularly clinically relevant.^[^ ^9^ ^]^

Titanium is a very common implant material as it has a lower rate of failure and fewer complications, compared to other metal materials like steel. However, titanium implants exhibit a higher propensity for tissue adhesion compared to their stainless steel counterparts.^[^ ^10^ ^]^ The tissue‐adhesive properties of titanium implants, which are beneficial for long‐term implants, may pose risks for the application of short‐term implants, potentially leading to reduced range of motion, joint stiffness, and, in severe cases, neurovascular complications due to removal. These adhesion‐related complications can result in protracted recovery periods, increased patient morbidity, and substantial psychological distress. Only a few articles deal with the question of how to use the biomechanical advantages of titanium for short‐term implants and modify them to avoid tissue adhesion and thereby escaping the negative side effects during or after implant removal.^[^ ^11^ ^]^ Furthermore, existing coatings for implants often lack robust covalent attachment to the titanium substrate, but instead rely on superficial application akin to wax deposition. In light of these challenges, the development of durable antiadhesive coatings for short‐term titanium implants represents a promising strategy to mitigate post‐implantation complications and reduce the risks associated with implant removal.

An additional medical complication presents the inherent risk of surgical site infection associated with implantation procedures often necessitates prolonged antibiotic therapy or revision surgery, imposing additional burdens on healthcare systems and patients alike. In fact, literature found with anti‐adhesive coatings of titanium alloys mostly regards anti‐adhesion of bacteria to avoid bacterial adhesion and infection at the implant site.^[^ ^12^, ^13^ ^]^ Many current approaches incorporate antibiotics, silver nanoparticles, or drug‐release systems to mitigate infection risks.^[^ ^14^ ^]^ This modification method potentially results in unstable coatings susceptible to removal or dissolution, compromising their efficacy in preventing cellular attachment. Moreover, leaching of the active agent, especially of antibiotics, may inadvertently promote bacterial resistance, induce adverse systemic effects, or fail to maintain constant antibacterial efficacy.^[^ ^15^ ^]^

To address the issues previously discussed, an ideal coating for short‐term titanium implants would consist of an antifouling hydrogel covalently bound to the substrate, incorporating a non‐leaching antibiotic motif. In this context, hydrogels derived from water‐swollen polymer networks have emerged as a particularly effective class of materials for preventing protein and cell adhesion.^[^ ^16^ ^]^ As such, they present an attractive solution to the challenges associated with short‐term implants, potentially acting as a protective barrier against the surrounding cellular environment. The initial step in achieving this goal involves the development of a hydrophilic polymer network capable of binding to titanium.

The literature reports a diverse array of compounds suitable for hydrogel polymer networks. While some are derived from natural sources such as polysaccharides.^[^ ^17^ ^]^ or polypeptides,^[^ ^18^ ^]^ synthetic polymers, including polyacrylamides,^[^ ^16^ ^]^ polyacrylates, and poly(2‐oxazoline)s,^[^ ^19^ ^]^ offer greater versatility. The advantage of synthetic polymers lies in the ability to tailor their properties through judicious selection of monomeric composition and subsequent modification reactions. This allows for precise control over characteristics such as chain length, molar mass, functional groups, and overall polymer architecture. To generate covalent polymer networks, two general strategies are applicable: the use of multifunctional crosslinker comonomers for in situ network formation during polymerization, or the activation of trigger‐sensitive moieties in pre‐formed single‐chain polymers to enable post‐polymerization crosslinking.^[^ ^20^ ^]^ The latter approach offers the advantage of processing the soluble single‐chain polymers into coatings before inducing network formation. Photocrosslinking presents a particularly convenient type of post‐polymerization crosslinking, enabling network generation from pre‐synthesized polymers through light irradiation, with azide.^[^ ^21^ ^]^ and benzophenone derivatives.^[^ ^22^ ^]^ being prominent examples of photocrosslinking groups. An additional prerequisite for the hydrogel coating is a strong covalent bonding to the titanium implant. Phosphonic acid groups have emerged as potent anchor units for robust attachment to oxidized titanium surfaces.^[^ ^23^ ^]^ Antibacterial features can be incorporated into the polymer network via physical embedding or chemical attachment of active agents. To prevent loss of the antibacterial function over time, covalent bonding of the active species to the gel structure or imparting intrinsic antibacterial motifs directly to the polymer backbone are effective strategies.

In this study, we present a novel approach that combines antiadhesive acrylamide‐based hydrogels with benzophenone photocrosslinkers possessing intrinsic antibacterial properties and phosphonic acid anchor groups specifically targeting titanium substrates. This particular design aims to address the multifaceted requirements of coatings for short‐term titanium implants, potentially offering a significant advancement in implant technology.

## 2 Experimental Section

### 2.1 Equipment, Methods and Chemicals

#### 2.1.1 Equipment and Methods

The reactions were performed under an inert gas atmosphere with dried glassware.

All ^1^H and ^13^C NMR spectra were recorded with a spectrometer from Jeol Type ECZ 500. The ^1^H NMR measurements were recorded at a frequency of 500 MHz and the ^13^C NMR measurements at 126 MHz at room temperature (≈20 °C). ^31^P NMR (17.5 MHz) spectra were recorded at 28.5 °C on a Spinsolve 43 Phosphorus Benchtop NMR (Magritek, Aachen, Germany) with an internal NMR device reference. The chemical shifts of the signals *δ* were specified in ppm. The peaks of the undeuterated solvents were used as an internal standard. All recorded spectra were analyzed with the software MestReNova. The following abbreviations were utilized to describe NMR peak patterns: s = singlet, d = doublet, t = triplet, and dd = doublet of doublets.

Molar mass distributions and dispersities of polymers were measured by the Polymer Standard Service (PSS) SECcurity GPC system Agilent Technologies 1260 infinity. For measuring of RAFT‐copolymers, the system was equipped with a PSS GRAM 100 Å column, and for measurement of FR‐copolymers, a Gram Linear M column was used. In both cases, a 10 µm particle size pre‐column was applied. A solution of DMAc with lithium bromide (*c* = 1 g l^−1^) was used as an eluent. For detection, an IR detector and a UV/Vis detector, adjusted to a wavelength of 280 nm, were applied. A sample volume of 20 µl was injected. A temperature of T ≈ 60 °C and a flow rate of 1 ml min^−1^ were adjusted. The calibration curves were generated with poly(methyl methacrylate)‐standards (PSS, Mainz), and the molar mass of the polymers was determined by the system software. Elemental analysis measurements were performed with the EA 3000 CHNS.

All cell culture plates (ultra‐low attachment plates) or flasks used were from Cornig Inc. (New York, USA).

Titanium discs were manufactured from commercially pure grade 4 titanium (ISO 5832‐2, Medartis, Basel, Switzerland). Discs measuring 14.5 mm in diameter/1.6 mm in thickness and 33.5 mm in diameter/1.6 mm in thickness were used in this study. The diameters were chosen to ensure that the discs would match the diameter of 6‐ and 24‐well plates.

The titanium substrates were pre‐treated with a Sicatech corona generator Uni‐System LF1.

For photocrosslinking, the UV Crosslinker Bio‐Link from LTF Labortechnik was used with a total energy of 8.6 J cm^−2^.

Modified titanium discs were analyzed by environmental scanning electron microscopy (Quanta 250 FEG, FEI Deutschland GmbH, Dreieich, Germany) in low vac mode at 130 Pa and 20 keV, equipped with a combination of LF, GSE, and ET BSE detectors. Additional EDX was carried out with an EDS1, Apollo XL‐SDD. The generated data was processed with the TEAMTM Enhanced V. 4.5 software.

For atomic force microscopy (AFM) experiments, tipless silicon FCL‐5 cantilever with a nominal spring constant of 0.12 N m^−1^ and a resonance frequency of 14 kHz (Applied NanoStructures, Inc., Mountain View, California, USA), Dry Borosilicate Glass Microspheres with a nominal diameter of 20 µm (Duke Standards, Fremont, California, USA), Norland Optical Adhesive 73 (Norland Products, Inc., Cranbury, New Jersey, USA), BLX‐254 UV chamber (LTF Labortechnik, Wasserburg, Germany) equipped with T‐8L UV light tubes for emission of UV light with a wavelength of 365 nm (Vilber Lourmat, Eberhardzell, Germany) and a TGT1 diffraction grating to visualize the bead‐modified AFM tip (NT‐MDT LLC, Moscow, Russia) were used. Scanning electron microscopy was performed using a CamScan CS24 electron microscope (Cambridge, UK). AFM measurements were performed using an MFP‐3D‐Bio atomic force microscope (Asylum Research, Santa Barbara, California, USA) and analyzed using Igor Pro 6.38B01 software (WaveMetrics, Lake Oswego, Oregon, USA). MilliQ‐water was received from a Merck Millipore Direct Q 8 system (Millipore, Schwalbach, Germany) with a resistivity of 18.2 MΩ cm, hereinafter referred to as water. Force measurements were performed in polystyrene petri dishes (Sarstedt AG & Co. KG, Nümbrecht, Germany).

EVOS Fluoreszenz–Microscope (Thermo Fisher Scientific Inc. Waltham, USA), Digital microscope, Keyence BZ‐X800 Series (Keyence Corporation of America Itasca, USA), Biological safety cabinets, NuAIRE, NU‐440‐600E, class II (NuAire Plymouth, USA), FACS (Cytoflex, Beckman Coulter, Brea, USA).

#### 2.1.2 Chemicals

*N*‐(2‐hydroxyethyl) acrylamide (HEAm, 97%‐Merck, Darmstadt, Germany), vinyl phosphonic acid (VPA, >= 90%‐Sigma Aldrich, Steinheim, Germany), trifluoroacetic acid (TFA, 99%‐Carl Roth, Karlsruhe, Germany), lithium bromide (LiBr, 98%‐Alfa Aesar, Haverhill, Massachusetts, USA), 2,2′‐azobis[2‐(2‐imidazolin‐2‐yl)propane] dihydrochloride (AIPC, 97%‐Waco Chemicals, Neuss, Germany), and Lewatit MP62 (Lanxess, Köln, Germany) were used as received. Azobisisobutyronitrile (AIBN ‐ Fluka, Charlotte, North Carolina, USA) and acrylamide (Am, Carl Roth, Karlsruhe, Germany) were both recrystallized from methanol twice. The solvents methanol (MeOH, 99.8%‐Fisher Scientific, Schwerte, Germany), ethyl acetate (EtOAc, 99.8%), ethanol (EtOH, 99+%), ethanol absolute (abs. EtOH, 99,9+%), and *N,N*‐dimethylacetamide (DMAc, 99.5% ‐all VWR, Darmstadt, Germany) were used without further purification. Deuterated solvents for NMR analysis (Deutero, Kastellaun, Germany) were used as received. MilliQ‐water was taken from a Merck Millipore system (σ = 18.2 MΩ cm, Millipore, Schwalbach, Germany).

#### 2.1.3 Cell Culture

Media for both cell cultures DMEM/F‐12 (1:1) (1X) + GlutaMAX‐I Dulbecco´s Modified Eagle Medium F‐12 Nutrient Mixture (Thermo Fisher, Waltham, USA) supplemented with 10% FCS (Biochrom GmbH, Berlin, Germany) and 1% Penicillin/Streptomycin (Sigma Aldrich, Steinheim, Germany). Tenocytes were isolated from waste material during surgery (see below), NHDF (normal human dermal fibroblasts) were purchased from Promocell (Heidelberg, Germany), L929 murine fibroblasts from ATCC (Manassas, USA), Accutase for cell detachment (Sigma Aldrich, Steinheim, Germany), for proliferation tests alamarBlue Cell Viability Reagent (Thermo Fisher, Waltham, USA), for cytotoxicity tests MTT (thiazolylblau‐tetrazoliumbromid; Sigma Aldrich, Steinheim, Germany).

Human tenocytes were isolated from surgical tendon material obtained during the reconstruction of the anterior cruciate ligament (ACL plastic). The use of residual materials was approved by the ethics committee of the Landesärztekammer Rheinland‐Pfalz in agreement with the university medical center and in accordance with the principles expressed in the Declaration of Helsinki and the ICH Guidelines for GCP. All patients provided written consent. First, connective tissue, fatty tissue, and other types of tissue that were not required were carefully separated from the tendon material using a scalpel and forceps so that only the tendon structure remained intact. The cleaned tendon material was then cut into sterile 2 mm pieces using a scalpel. Depending on the size of the tendon, ≈10–20 pieces were prepared and transferred to a Petri dish and placed in cell culture medium containing ascorbic acid at a concentration of 50 µg ml^−1^. Tenocytes grew out of the pieces and were approved to be tenocytes by fluorescent staining of typical markers.

#### 2.1.4 Microbiology Testing

Müller–Hinton–Agar (Thermo Fisher, Waltham, USA), BD Trypticase Soja–Agar (Becton Dickinson GmbH, New Jersey, USA), Mc Farland BSS 0.5 standard (Carl Roth GmbH + Co. KG, Karlsruhe, Germany), Staphylococcus aureus Culti–Loops subsp. ATCC 29213 (Fisher Scientific GmbH, Dreieich, Germany)

### 2.2 Synthesis Procedures

#### 2.2.1 Main Monomers

The main monomer *N*‐(2‐hydroxypropyl) acrylamide (HPAm) was prepared as reported elsewhere.^[^ ^24^ ^]^ The acidic acid‐based monomer *N*‐(2‐acetic acid) acrylamide (AaAm) was synthesized according to a published procedure.^[^ ^25^ ^]^ The Boc‐protected *N*‐(2‐aminoethyl) acrylamide (AEAm‐Boc) was synthesized in a two‐step reaction, according to the literature.^[^ ^26^ ^]^ The Boc‐protected *N*‐(3‐aminopropyl) acrylamide (APAm‐Boc) was synthesized in a two‐step reaction. In the first step, the single Boc‐protected 1,3‐diaminopropane (*N*‐tert‐butoxycarbonyl‐1,3‐diaminopropane) was synthesized according to a literature procedure.^[^ ^27^ ^]^ In the second step, *N*‐tert‐butoxycarbonyl‐1,3‐diaminopropane was modified with acryloyl chloride to yield the corresponding vinylic monomer. The reaction was performed in analogy to the literature.^[^ ^26^ ^]^

#### 2.2.2 Crosslinker Monomers

The syntheses of the acrylamide‐based crosslinker monomers *N*‐(4‐benzophenyl)acrylamide (BPAm),^[^ ^28^, ^29^, ^30^ ^]^ and [(4‐benzoylphenyl)methyl]dimethyl[3‐(prop‐2‐enamido)propyl]azanium bromide (BPQAAm).^[^ ^31^ ^]^ were performed according to literature.

#### 2.2.3 Adhesion Monomers

Brij35 acrylate (B35A), was synthesized according to literature.^[^ ^32^ ^]^

#### 2.2.4 Chain Transfer Agents

The CTA 2‐(dodecylthiocarbonothioylthio)‐2‐methylpropionic acid (DMP) was prepared in analogy to procedures published elsewhere.^[^ ^33^ ^]^

#### 2.2.5 Adhesion Polymer

The synthesis of the adhesion polymer poly(Am_93_‐*co*‐BPAm_2_‐*co*‐B35A_5_) was performed according to literature.^[^ ^32^ ^]^

#### 2.2.6 Anchor Polymers

Anchor polymers were synthesized via free radical polymerization according to the following general procedure. Vinyl phosphonic acid (VPA), *N*‐(2‐hydroxyethyl) acrylamide (HEAm), and 2,2′‐azobis(isobutyronitrile) (AIBN) were dissolved in methanol (total volume *V*_total_ = 5 ml) and the solutions purged with argon for 30 min while stirring. The solutions were placed in a pre‐heated aluminum block (65 °C) and the reaction took place for 21 h. The reaction time was reached, and the polymerizations were quenched by cooling with liquid nitrogen and exposure to air. The polymers were precipitated in ice‐cold ethyl acetate (120 ml), filtered off, and dried in a vacuum overnight. For specific amounts of educts, yields, and GPC analysis results, see Table S1 (Supporting Information).

#### 2.2.7 Co‐ and Terpolymers for Double Layer Coatings

For the preparation of double‐layer coatings, 17 different polymers were synthesized via FR or RAFT polymerization. For each polymer, the monomers, initiator, and, in the case of RAFT polymerization, the CTA DMP were dissolved in methanol (*V*_total_ = 2‐5 ml). The reaction mixtures were purged with argon for 30 min and placed in a preheated oil bath or aluminum block (65 °C) for 16 to 24 h. After completion, the polymerizations were quenched by cooling with liquid nitrogen and exposure to air. All polymers were precipitated in ice‐cooled ethyl acetate (200 ml) at least twice. The polymers were separated by centrifugation or filtration and dried under reduced pressure overnight. The specific amounts of educts, yields, and GPC analysis results are summarized in Tables S2 and S4 (Supporting Information).

#### 2.2.8 Deprotection of Polymers Containing Boc‐Protected Amino Groups

In a dry 10 ml Schlenk tube typically 50–100 mg of the of polymers containing Boc‐protected amino groups were dissolved in water (1.5 ml). To the mixture, TFA (0.5 ml) was added and the mixture was stirred at room temperature overnight. The polymers were precipitated in ice‐cold THF. The precipitate was then redissolved in water and stirred with cationic ion exchange resin (Lewatit MP62) for 24 h. The resin was finally filtered off and the filtrate lyophilized. For specific amounts of educts, yields, and GPC analysis results, see Table S3 (Supporting Information).

### 2.3 Coating Procedures

#### 2.3.1 Coating of Poly(styrene) Well Plates

Aliquots of an adhesion polymer solution (70 µl for full coatings or one or two droplets for partial coatings, water/ethanol mixture *V*/*V* = 7:3, 2.5 mg ml^−1^) were added in each well of a 24‐well plate and the plate dried under reduced pressure overnight at *T* = 40 °C. The dried polymer films were irradiated with UV‐light (*λ* = 254 nm) for 1 h. Afterward, the hydrogel precursor polymer stock solutions (200 µl for full coatings 10 µl for partial coatings, water/ethanol mixture *V*/*V* = 7:3, 25 mg ml^−1^) were deposited on top of the adhesion polymer film and dried in vacuo overnight at *T* = 40 °C. The dried films were irradiated with UV‐light (*λ* = 254 nm) for 1 h. The polymer networks were washed by swelling in water for at least 1 h, rinsed with ethanol, and dried in vacuum at *T* = 40 °C.

#### 2.3.2 Coating of Titanium Substrates

The circular titanium substrates were pre‐cleaned by scrubbing with detergent (akuta by Dalli Werke, Stolberg, Germany) and rinsing with water. Subsequently, the substrates were exposed to a Hellmanex (VWR, Darmstadt, Germany) cleaning solution (3%) overnight at room temperature. The cleaned disks were rinsed with water and ethanol, dried in a nitrogen stream, and treated with a corona discharger for ≈10 s. Directly after the treatment, the titanium disks were immersed into water. For full coatings, the pre‐treated titanium disks were immersed in an aqueous anchor polymer solution (1 mg ml^−1^) overnight. For partial coatings, the pre‐treated titanium disks were immersed halfway in an aqueous anchor polymer solution (1 mg ml^−1^) overnight, by placing them upright into a suitable container. The titanium disks were then rinsed with water and ethanol, placed into 24 well plates, and dried under reduced pressure at *T* = 40 °C for 2 h. Afterward, the hydrogel precursor polymer stock solutions (200 µl for full coatings or 100 µl for partial coatings, water/ethanol mixture, *V*/*V* = 7:3, 25 mg ml^−1^) were deposited on top of the anchor polymer film and dried in vacuo overnight at *T* = 40 °C. The dried films were irradiated with UV‐light (*λ* = 254 nm) for 1 h. After completing crosslinking, the polymer networks were washed by swelling in water for at least 1 h, rinsed with ethanol, and dried in a vacuum at *T* = 40 °C.

### 2.4 Analyses

#### 2.4.1 AFM Measurements

Force measurements were carried out on the hydrogel by measuring single force curves in air and force maps of 90 µm size with 100 force curves per force map in water. Individual force curves were analyzed by applying the software‐implemented algorithm for offset correction to determine the point of contact and then applying the Hertz model to fit the approach curve from 0 to 40% of indentation depth. The radius of the indenter was assumed to be a spherical indenter with a radius of 10 µm having a Young´s modulus of 68 GPa and a Poisson ratio of 0.19 as defined in the software. For the sample, a Poisson ratio of 0.33 was assumed, according to the literature.^[^ ^34^ ^]^

### 2.5 In Vitro Tests

#### 2.5.1 Cytotoxicity Assay

In vitro cytotoxicity was analyzed analogously to ISO 10993‐5 using the MTT ([3‐(4,5‐dimethylthiazol‐2‐yl)‐ 2,5‐diphenyltetrazoliumbromid)]) assay. Mouse L929 cells (10000 cells well^−1^) were seeded in a 96‐well tissue culture plate for 24 h. Polymers either on 24 cell culture plates or titanium discs in 24 well plates were incubated in 1 mL cell media for 24 h. ≈100 µL of this extract was given to L929 cells in the 96‐well plate. After an incubation time of 24 h, the MTT assay was performed, and the colorimetric readout was performed at a wavelength of 570 nm (reference wavelength 650 nm). Zinc diethyl dithiocarbamate (ZDEC) and zinc dibutyl dithiocarbamate (ZDBC) (Food and Drug Safety Center, Hatano Research Institute, Hadano, Japan) were used as positive controls as they induce a reproducible cytotoxic reaction.

#### 2.5.2 Lentiviral Transduction

Gene transfer of eGFP into NDHF and tenocytes was achieved by lentiviral transduction. The eGFP encoding lentiviral vector pHR0‐SEW was used to prepare vector supernatants by transfection of 293T cells as previously described.^[^ ^11^, ^35^ ^]^ For gene transfer, 15.000 cells were seeded into 24‐well tissue culture plates. Two rounds of transduction on days 1 and 3 were performed at a cumulative multiplicity of infection (MOI) of *100 to achieve 90–95% gene marking. Transduction efficiency was confirmed by flow cytometry.

#### 2.5.3 Adhesion Assays

For the investigation of cell adhesion and cell growth, 20 000 fibroblasts or tenocytes were seeded per well/titanium disc. The volume calculated for 20000 cells was pipetted onto the titanium discs or the coated well and as a control on uncoated cell culture wells. The fluorescence microscopic examination of cell growth and cell adhesion was carried out on day 1, day 3, and day 7 after sowing. The images were taken according to a standardized scheme to facilitate comparison of the images.

On the monitoring days, overview images were taken at magnifications of 2x and 4x as well as detailed images at a magnification of 10x. The image sections of the overview images were based on the following areas: I. Upper outer edge area, II. right outer edge area, III. lower outer edge area, IV. Left outer rim area and V. Centre. At least 3 experiments were carried out for each coated cell culture well or titanium disc.

In order to test whether the non‐adherent cells were still viable, they were transferred to a culture well to test if they were still able to adhere and proliferate after contact with the polymer.

#### 2.5.4 Microbial Testing

The polymers used were analyzed for an intrinsic antimicrobial effect. The bacterium Staphylococcus aureus was used to demonstrate this effect. Staphylococcus aureus was cultured and afterward diluted to a turbidity corresponding to McFarland Standard 4. Subsequently, 2 µl of this bacterial suspension and 1000 µl of culture medium were pipetted onto the coated titanium discs incubated at 37 °C for 24 h. After incubation, on the one hand, OD_600_ was measured and on the other hand, the contaminated medium was plated on Mueller–Hinton culture media, and after 24 h colonies were documented photographically after spreading in order to record bacterial growth.

## 3 Results and Discussion

This chapter details the multi‐step development process for effective hydrogel coatings on short‐term titanium implants. First, the synthesis and evaluation of the monomer and polymer components are discussed, as these directly impact the hydrogel's key characteristics. The coating procedure is then described, outlining the methods for achieving uniform surface coverage on the titanium substrates. Finally, the biological performance of the coatings is evaluated through in vitro assessment of cellular response and bacterial colonization.

### 3.1 Monomer and Polymer Synthesis

**Scheme 1:**
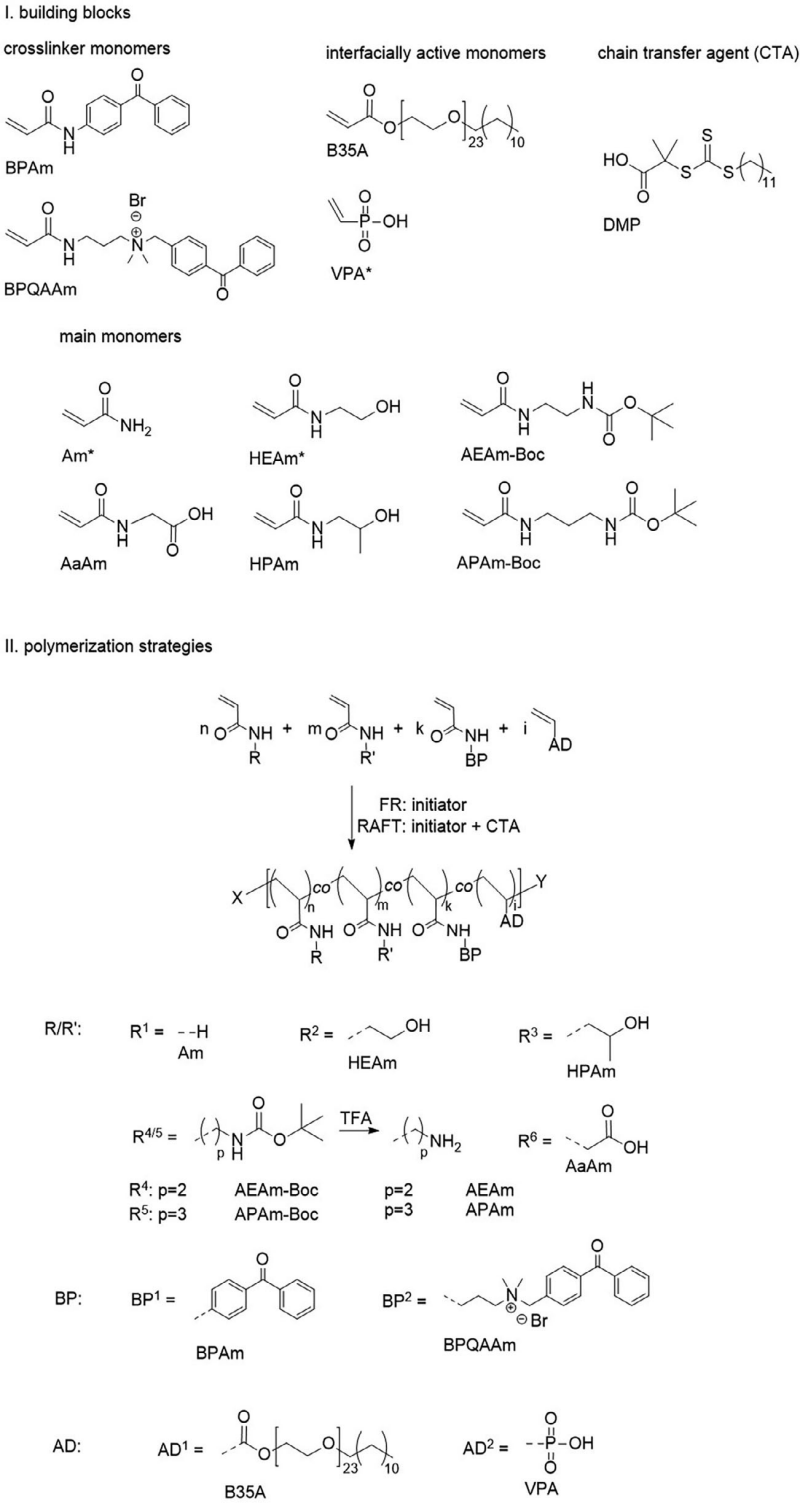
Structures of the building blocks and synthesis strategies for the antifouling polymers, with AD = adhesion promoting group, BP = benzophenone crosslinker, and R/R’ = main comonomer units (* commercially available compounds).

An overview of the building blocks, which are divided into crosslinker monomers, interface‐active monomers, main monomers, and the chain transfer agent, is given in **Scheme** **1**, part I.

#### 3.1.1 Monomer Syntheses

The coatings for the short‐term titanium implants were developed using a variety of monomers with different functionalities. The first category comprises the crosslinker monomers, which have a double bond for polymerization and a photoreactive benzophenone unit to enable crosslinking between polymer chains upon irradiation. Two different crosslinker monomers were synthesized and evaluated. The first, BPAm, is a well‐established photocrosslinker monomer.^[^ ^36^ ^]^ The synthesis of BPAm was performed following literature procedures,^[^ ^28^, ^29^, ^30^ ^]^ Briefly, 4‐aminobenzophenone was exposed to an excess of acryloyl chloride, forming the BPAm monomer with a yield of 88%. However, BPAm has poor solubility in aqueous media, which would not be ideal for the biological environment surrounding the implants. Good solubility in water‐based bodily fluids would be more beneficial for this application. The second photocrosslinker, BPQAAm, has a similar structure to BPAm but includes a quaternary ammonium unit in the spacer between the photoreactive and polymerizable moieties. The permanently positive charge of the ammonium group provides higher affinity toward water and is a common motif in antibacterial agents.^[^ ^37^ ^]^ BPQAAm was synthesized in a two‐step reaction sequence as previously published.^[^ ^31^ ^]^ In the first step, bromination of 4‐ methylbenzophenone with *N*‐bromosuccinimide and azobisisobutyronitrile (AIBN) was performed, yielding 84% of 4‐bromomethyl benzophenone. In the second step, *N*‐[3‐ (dimethylamino)propyl]acrylamide was quaternized with 4‐bromomethyl benzophenone to yield 90% of the final product BPQAAm.

Another monomer type is the interface‐active monomers, which promote adhesion to specific surfaces. The amphiphilic surfactant monomer B35A, with a hydrophobic alkyl chain, was used for less polar polymeric surfaces like polystyrene well plates, where the alkyl chains can adsorb through hydrophobic interactions. B35A was synthesized from the surfactant Brij 35 in 91% yield by substituting the terminal OH group with acryloyl chloride under basic conditions, according to the literature.^[^ ^25^ ^]^ For the oxidized titanium surface, the commercially available vinyl phosphonic acid (VPA) was employed as anchor group.

The main monomers serve as the foundation for the hydrophilic polymer networks. Their highly hydrophilic characteristics complement the specific functions of the other monomer types, embedding them in an environment well‐suited for strong interactions with aqueous solutions. The well‐established hydrophilic monomers acrylamide (Am) and hydroxyethyl acrylamide (HEAm) were obtained from commercial suppliers, while the additional main monomers 2‐hydroxypropyl acrylamide (HPAm).^[^ ^24^ ^]^ and the acrylamide of glycine (AaAm).^[^ ^25^ ^]^ were synthesized following published procedures. The synthesis of HPAm involved an exothermic reaction of 1‐amino‐2‐propanol with acryloyl chloride, yielding 30% of the target monomer. For AaAm, a reaction between glycine and acryloyl chloride under basic conditions resulted in an 11% yield. Additionally, the Boc‐protected monomers *N*‐(2‐aminoethyl)acrylamide‐Boc (AEAm‐Boc).^[^ ^26^ ^]^ and *N*‐(3‐aminopropyl)acrylamide‐Boc (APAm‐Boc).^[^ ^26^, ^27^ ^]^ were synthesized in a two‐step reaction sequence. The first step involved the reaction of the corresponding diamine with di‐*tert*‐butyl dicarbonate yielding 80% and 41% of the mono‐Boc‐functionalized diamine precursors, respectively. This step was followed by the conversion of the mono‐Boc‐functionalized diamines to the acrylamides using acryloyl chloride under mild basic conditions, resulting in 73% of AEAm‐Boc and 94% of APAm‐Boc. In addition to their role as a hydrophilic matrix base, the monomers AaAm, AEAm‐Boc, and APAm‐Boc offer the potential for post‐polymerization modification with specific functional groups.

#### 3.1.2 Polymer Syntheses

Two different polymerization methods were explored for the polymer structures shown in Scheme 1, part II: free radical polymerization (FRP) and reversible addition‐fragmentation chain transfer polymerization (RAFT polymerization). FRP is easily implemented yet allows less control, leading to high molar masses with a broad distribution.^[^ ^38^ ^]^ In contrast, RAFT polymerization provides a higher degree of control by employing chain transfer agents (CTAs) to mediate the interchange of active radical sites at the chain ends. This allows to target the molar mass of the polymer with dispersities *Ð* as low as 1.2.^[^ ^38^ ^]^ The CTA fragment remains as an end group in the RAFT polymers, which is in contrast to the FRP polymers that feature chain ends resulting from the initiator and termination reaction. All structures of the synthesized polymers can be found in the overview graphic (see Figure S1, Supporting Information). The effect of the structural difference in both polymer types on their performance as antiadhesive and antibacterial coatings was evaluated in in vitro with cells and bacteria, as described in the biological experiments further below.

DMP as CTA for the RAFT polymerizations was obtained with a yield of 39% in a one‐pot reaction according to the literature.^[^ ^33^ ^]^ by first adding carbon disulfide to a solution of *n*‐dodecanethiol and triethylamine, followed by reaction with α‐bromoisobutyric acid.

The adhesion promoter polymer poly(Am‐*co*‐B35A‐*co*‐BPAm) (**AP0**, Figure S1, Supporting Information) provides covalent attachment of the hydrogel precursor polymers to polystyrene well plates and was prepared according to the literature.^[^ ^32^ ^]^ via FRP of acrylamide, the surfactant monomer B35A, and the crosslinker monomer BPAm with a yield of 83%.

For attachment to titanium surfaces, a variety of phosphonic acid‐containing anchor polymers (**AP1**‐**AP4**, Figure S1, Supporting Information) were synthesized. Vinyl phosphonic acid (VPA) and hydroxyethyl acrylamide (HEAm) were copolymerized in varying ratios (2, 5, 10, and 20 mol% VPA) using AIBN as initiator. The GPC results indicate a rapid increase in dispersity *Ð* with higher VPA content, from 1.6 for 2 mol% VPA to 3.0 for 20 mol% VPA, in line with literature reports.^[^ ^39^ ^]^ However, the molar masses in the presence of VPA (30–34 kg mol^−1^) matched the HEAm homopolymer equivalent prepared by FRP (32 kg mol^−1^). It was challenging to determine the actual VPA content incorporated into the copolymers, as the ^1^H NMR signals of the HEAm and VPA‐based repeat units overlap, precluding quantitative analysis. However, the ^31^P NMR spectra qualitatively show an increase in phosphorus content with higher VPA monomer feed, suggesting successful copolymerization.

A broad range of hydrogel precursor polymers (**A** – **P**, Figure S1, Supporting Information) were synthesized via both FRP and RAFT polymerization (see Tables S2–S4, Supporting Information). The reactions typically yielded 65–98% of the target polymers. The RAFT‐derived polymers showed molar masses from 11 to 39 kg mol^−1^, fluctuating around the target mass of 25 kg mol^−1^, with low dispersities from 1.2 to 1.4. In contrast, the FRP products had higher molar masses (≈40–70 kg mol^−1^) and broader dispersities between 2.2 and 3.8, as expected for uncontrolled polymerizations.^[^ ^38^ ^]^ For the APAm‐Boc and AEAm‐Boc‐containing polymers **A** and **B**, the Boc protecting groups were cleaved by reaction with TFA, and treated with a basic ion exchanger to yield the free amino side groups. This deprotection step resulted in a reduction of the apparent molar masses in GPC, likely due to the loss of the Boc group affecting the polymer hydrophilicity and consequently the hydrodynamic radii.

### 3.2 Coatings on Polystyrene and Titanium Substrates

The efficacy of the prepared polymers as hydrogel coatings in terms of antiadhesion was evaluated by modifying sterile tissue‐grade polystyrene well plates with the polymers. Two different setups were employed: fully coated wells and partially coated wells. The partially coated wells were used to determine if the cells would remain viable and adhere near the coated area on the well plate bottom. The fully coated wells were specifically used for the toxicity and cell viability tests.

#### 3.2.1 Coatings on Polystyrene Well Plates

The coating procedure involved first applying an adhesion polymer layer by dissolving the respective polymer in a water and ethanol mixture and adding it to the well. This layer was then dried and crosslinked using UV light. Subsequently, a layer of the hydrogel precursor polymer was added by applying a solution of the polymer on top of the adhesion polymer layer. This layer was then dried, crosslinked, and washed by adding water and allowing it to sit for 1 h. Finally, the wells were cleaned with ethanol and dried before performing the cell and bacteria tests.

#### 3.2.2 Coatings on Titanium Disks

**Figure 1:**
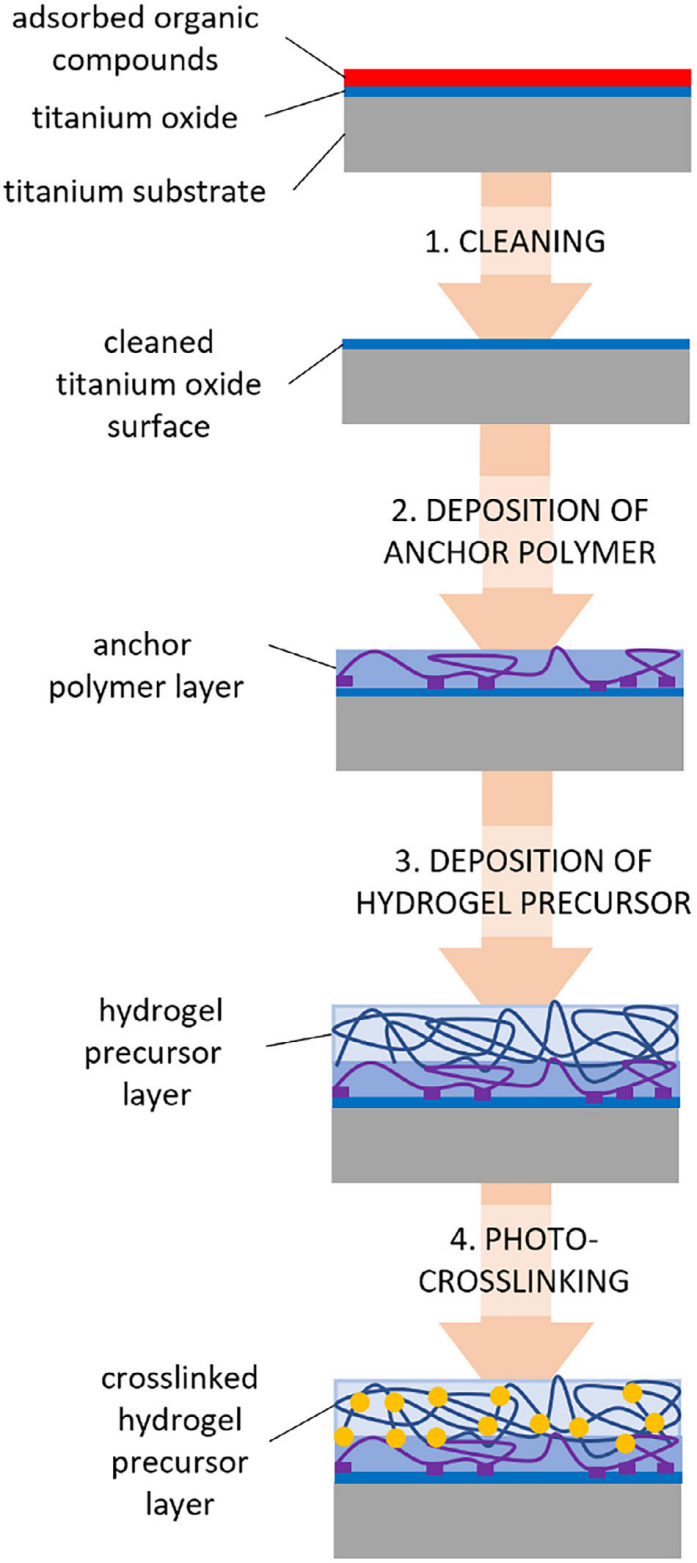
Schematic illustration of the process to generate a multi‐layer coating on titanium substrates. The process involves the deposition of an anchor polymer layer, followed by the deposition of a hydrogel precursor layer and photocrosslinking inducing network formation. When implanted, this network absorbs water from the surrounding tissue to swell into the antiadhesive hydrogel coating.

For the coating of titanium substrates, the above‐described anchor polymers **AP1** – **AP4** (see Figure S1, Supporting Information) were developed. The circular titanium disks used as the substrate were prepared for the coating procedure by scrubbing with detergent, followed by rinsing with water and incubation in an alkaline cleaning solution. To activate the surface, the disks were exposed to a corona discharge for several seconds and placed directly into water to generate OH groups on the titanium surface. The cleaned and activated titanium disks were incubated in an anchor polymer solution overnight, rinsed with water and ethanol, and dried. The hydrogel precursor polymer was then dissolved and added to the disk, which was placed inside a well plate. The precursor polymer layer was dried, crosslinked with UV light, and washed by exposure to water. Finally, the disks were rinsed with ethanol, dried, and used for the in vitro tests, discussed further below. A workflow of the titanium sample preparation is shown in **Figure** **1**.

#### 3.2.3 Contact Angle Measurements

The effect of the modification steps on the titanium surface was analyzed by measuring the static and dynamic water contact angles (CAs) for titanium disks as received, after cleaning, and with the anchor polymer coatings. The results (see Figures S8 and S9, Supporting Information) show that the as‐received titanium disks had relatively high water contact angles, ranging from 68° for static CA to ≈90° for dynamic CA. These comparably high CAs are rather unexpected for the polar titanium oxide material found on titanium under ambient conditions and suggest adsorbates that contaminate the surface. After applying the aforementioned cleaning procedure, the contact angles decreased significantly to 36° static CA and ≈45° dynamic CA. After application of the anchor polymer layer containing 10% VPA, even lower contact angle values of 29° static CA and 25° dynamic CA were observed. These findings indicate successful modification of the titanium surface with the anchor polymers and demonstrate the increased hydrophilicity of the coated substrates, facilitating the spreading of the aqueous hydrogel precursor solutions. As the titanium substrates with the hydrogel coating comprising the anchor polymer and the crosslinked hydrogel precursor were swollen and completely wetted by water, contact angles were too low to be measured.

#### 3.2.4 ESEM‐EDX Measurements

**Figure 2:**
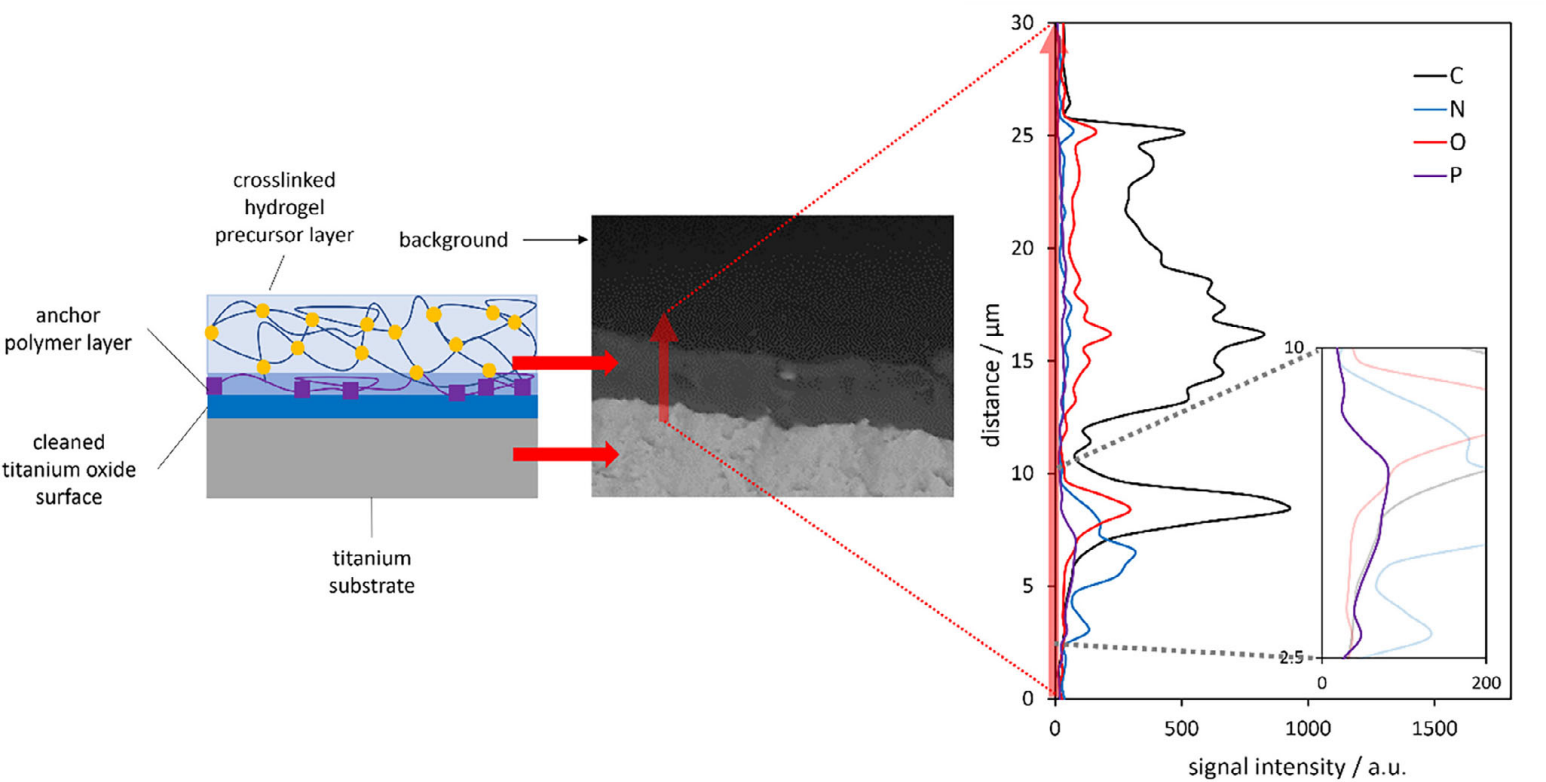
Environmental scanning electron microscopy (ESEM) image of the cross‐section of a coated titanium substrate cut in half. The red arrow indicates the direction of the line scan for the EDX analysis shown on the right‐hand side. The insert highlights the trace of the phosphorous signal (violet) from the anchor polymer layer.

To corroborate the two‐layer architecture of the polymer network coating, environmental scanning electron microscopy (ESEM) coupled with energy‐dispersive X‐ray (EDX) spectroscopy was utilized. **Figure** **2** shows the results of this measurement for a polymer‐coated titanium sheet, which was cooled with liquid nitrogen and cut in half to expose the cross‐section. The ESEM‐EDX analysis of the cross‐section revealed the expected sandwich structure of the coating, with the carbon signal (black trace) following the position of the overall organic layers. A distinct peak in the phosphorus signal (violet trace) at the interface between the titanium substrate and the hydrogel precursor layer indicates the successful binding of the phosphonic acid‐containing anchor polymer to the titanium surface.

#### 3.2.5 AFM Measurements

The mechanical properties of the swollen hydrogel coatings on the titanium substrates were investigated using atomic force microscopy (AFM). For this purpose, the hydrogel layer was indented by a spherical tip with a diameter of ≈25 µm mounted on a cantilever with a spring constant of 250 ± 30 pN nm^−1^ (for further details see Figures S12–S17, Supporting Information). From these measurements, Young's modulus of the hydrogel coatings was determined by a Hertzian model with a maximum value of 10.3 kPa and a full width at half maximum of 11.2 kPa, as derived from a Gaussian fit. These values are in good agreement with the typical range of 0.2 kPa to 50 kPa reported for Young's moduli of swollen hydrogels, depending on the degree of crosslinking.^[^ ^40^, ^41^ ^]^

From optical inspection and morphological analysis with the above methods (ESEM and AFM) a homogeneous surface coating with the polymers was confirmed (see Figures S18–S21, Supporting Information).

### 3.3 In Vitro Experiments

#### 3.3.1 Cytotoxicity Tests

**Figure 3:**
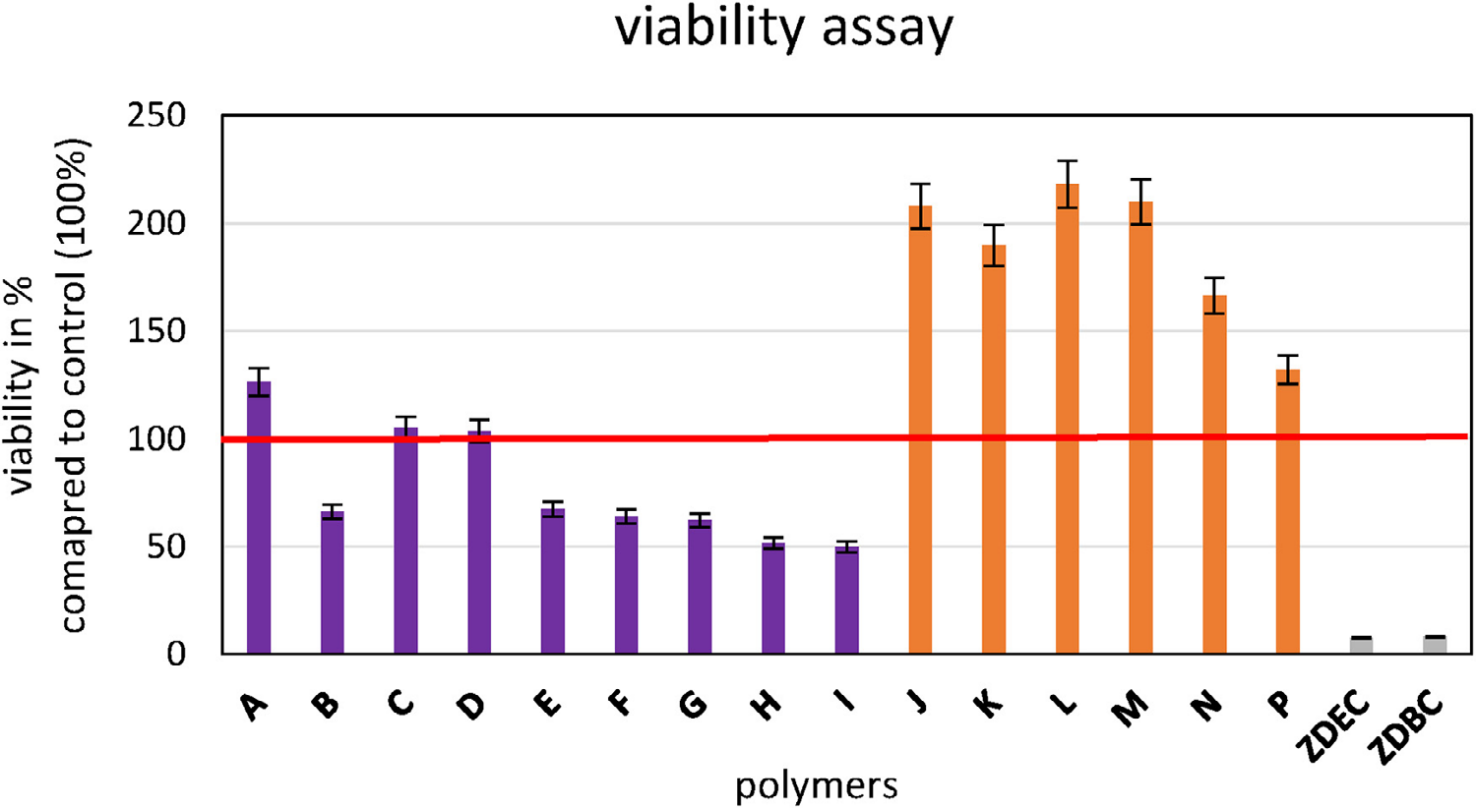
MTT tests performed analogous to ISO 10993‐5 show the different effects of the tested polymers. The red line represents the viability with normal cultivation medium (100%); ZDEC and ZDBC: cytotoxic controls. Polymers synthesized by RAFT polymerization are shown in purple, while FRP‐derived polymers are highlighted in orange.

Before proceeding with cell and bacterial tests, the cytotoxicity of the polymers was evaluated following the DIN ISO 10993‐5 standard (Biological Evaluation of Medical Devices‐Tests for In Vitro Cytotoxicity). The results revealed cell viability ranging from 50 to 220%, indicating varying effects of the polymers on the cells (see **Figure** **3**). Materials with cell viability below 70% were deemed non‐biocompatible and excluded from further investigation in cell culture and bacterial experiments.

**Figure 4:**
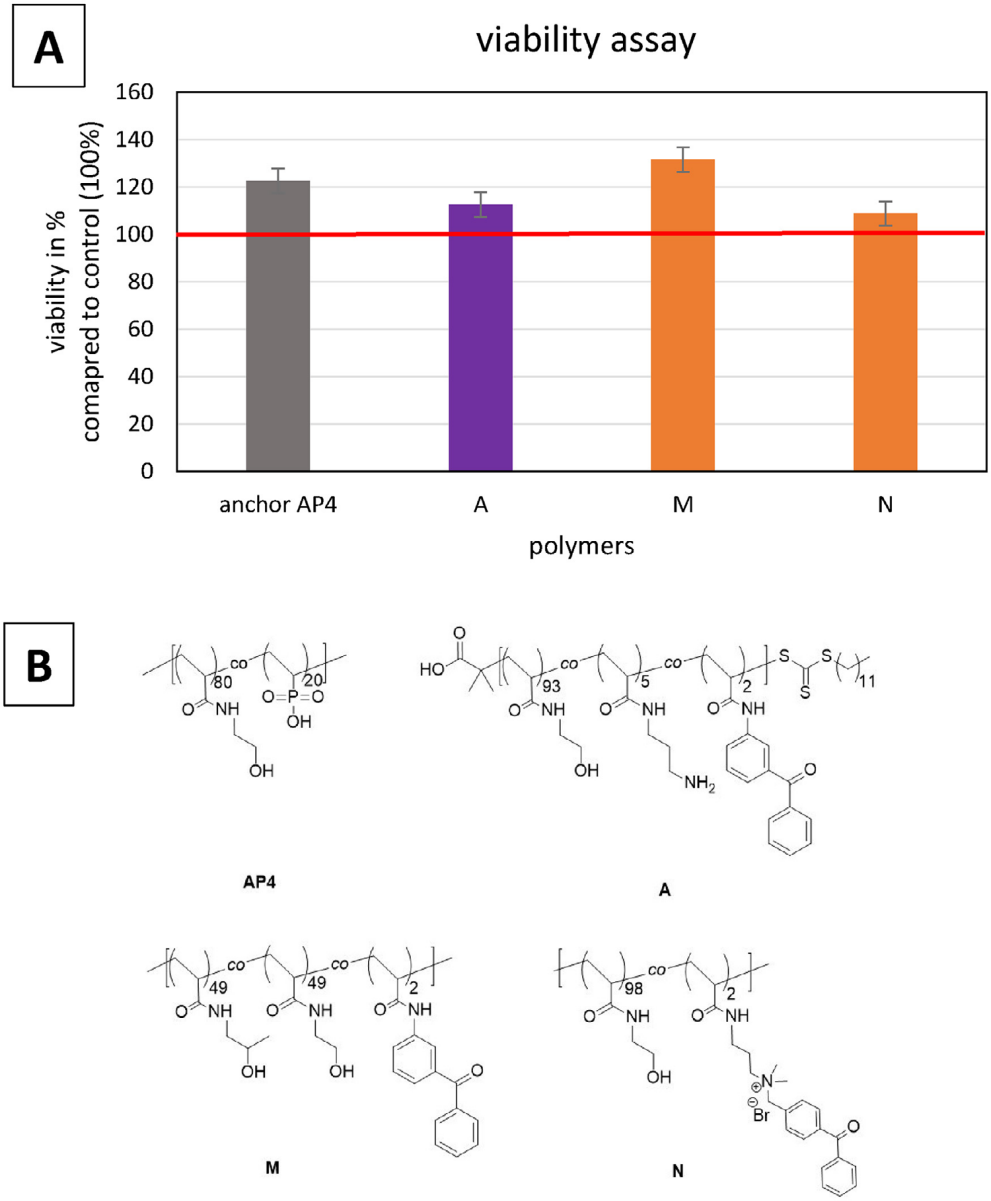
(A) MTT tests performed analogous to ISO 10993‐5 show the different effects of the tested polymers. The red line represents the viability with a normal cultivation medium (100%). RAFT polymers are shown in purple, while FRP‐derived polymers are colored in orange. (B) Structures of the polymers with the best performances.

The polymers **A** to **I** contain the end groups from RAFT polymerization and are more cytotoxic than the polymers **J** to **P** prepared by free radical polymerization. To reduce the number of tests to be performed on titanium substrates, the three best‐performing polymers from different classes were selected: **A** was chosen from the RAFT polymers with the lowest cytotoxicity from this group, **M** from the class of free radical polymerization, and **N** from free radical polymerization additionally including an antibacterial motif. As those polymers do not contain an anchor group for the titanium substrate, the titanium surface was first treated with a polymer containing a phosphonium anchor motif. As a first biological evaluation, the biocompatibility of the combined coating was confirmed (**Figure** **4**).

#### 3.3.2 Adhesion Tests

**Figure 5:**
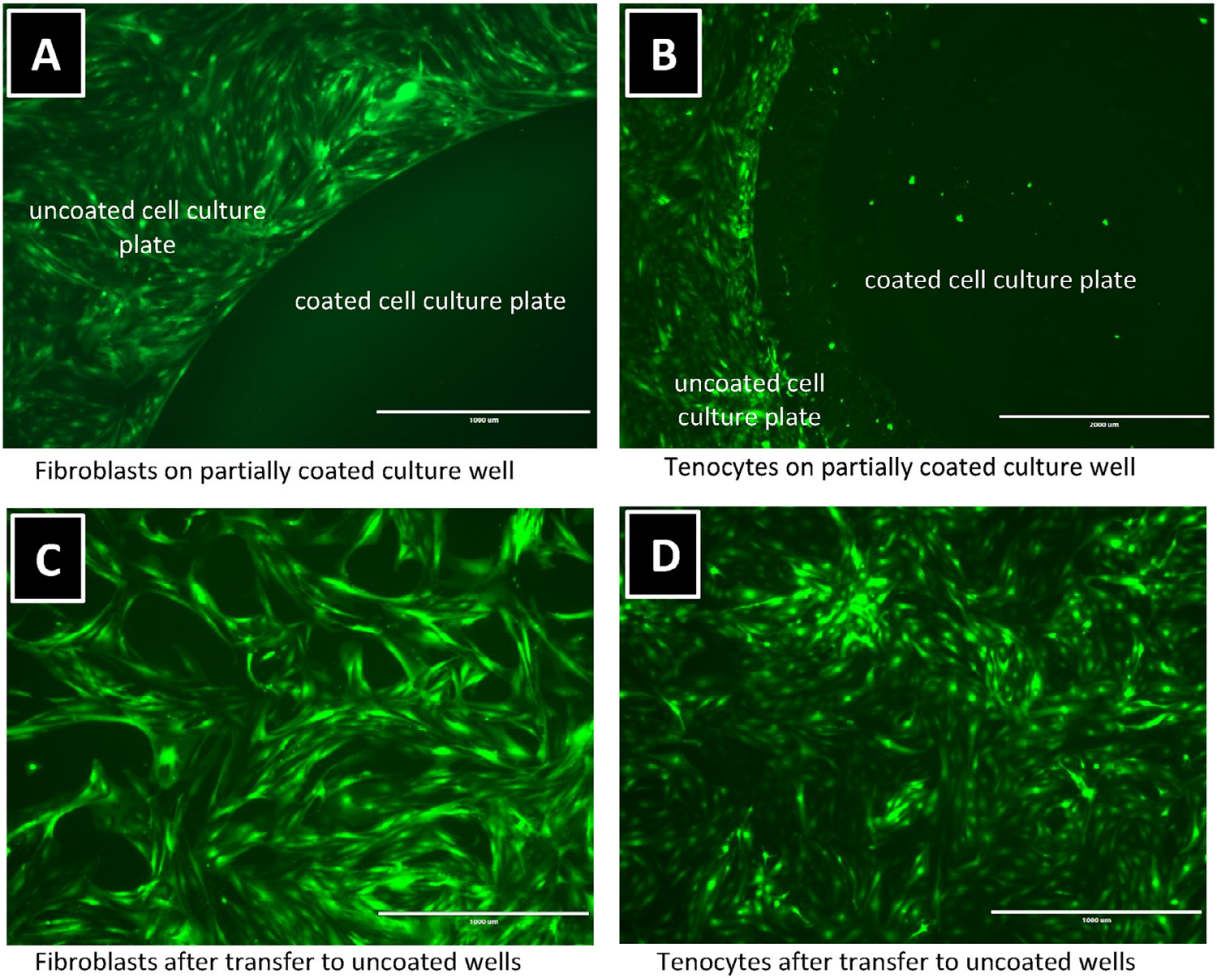
(A) Fibroblasts and (B) tenocytes seeded on partially coated cell culture plates with polymer N and after transfer to uncoated plates with (C) fibroblasts and (D) tenocytes. Similar observations were obtained with the polymers A and M (data not shown).

Primary fibroblasts and tenocytes were seeded onto cell culture plates partially coated with the polymer. As illustrated in **Figure** **5**, using tenocytes and the most promising polymer **N** as an example, the cells exclusively adhered to the uncoated areas of the plate. No cell adhesion occurred on the polymer‐coated regions. Similarly, no adhesion was observed on fully coated plates. However, the non‐adherent cells remained viable, as demonstrated by their ability to adhere and proliferate after being transferred to a standard cell culture plate.

**Figure 6:**
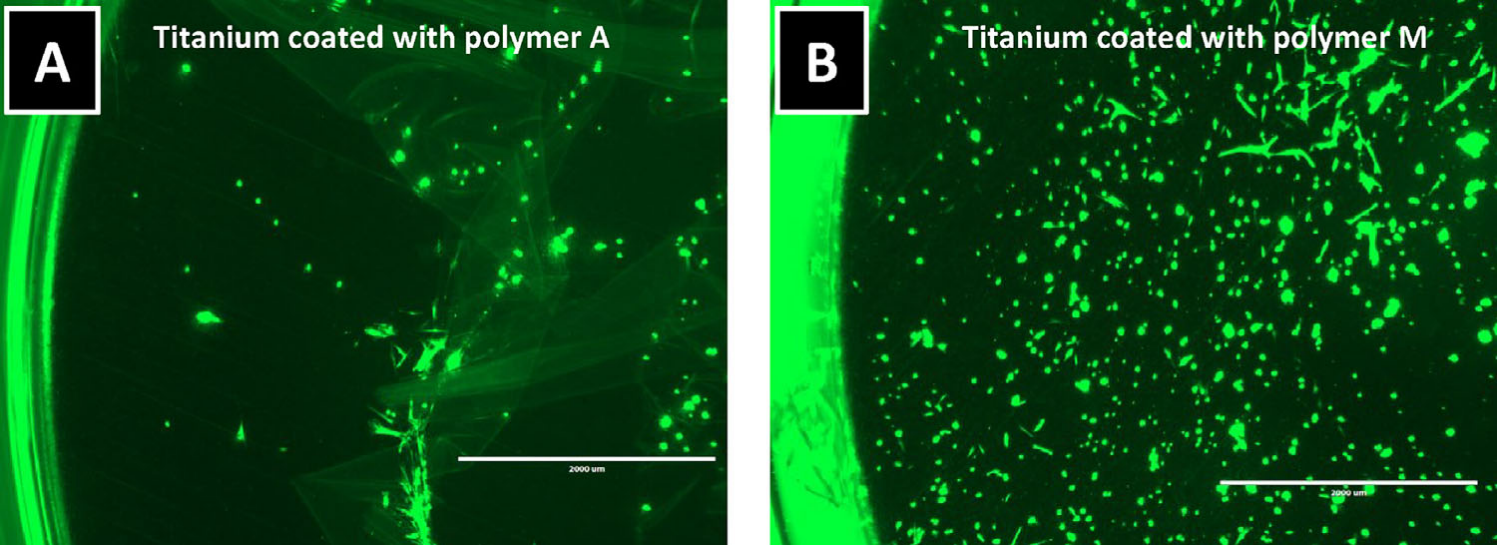
Fibroblasts seeded on fully coated titanium discs coated with (A) polymer A and (B) polymer M.

Polymers **A**, **M**, and **N** were subsequently applied to fully coated titanium discs. **Figure** **6** illustrates the discs coated with polymers **A** and **M**. On these surfaces, cells not only adhered to the coated areas but also, in the case of polymer **A**, the coating dissolved from the titanium surface. Due to these findings, both polymers **A** and **M** were excluded from further analysis.

**Figure 7:**
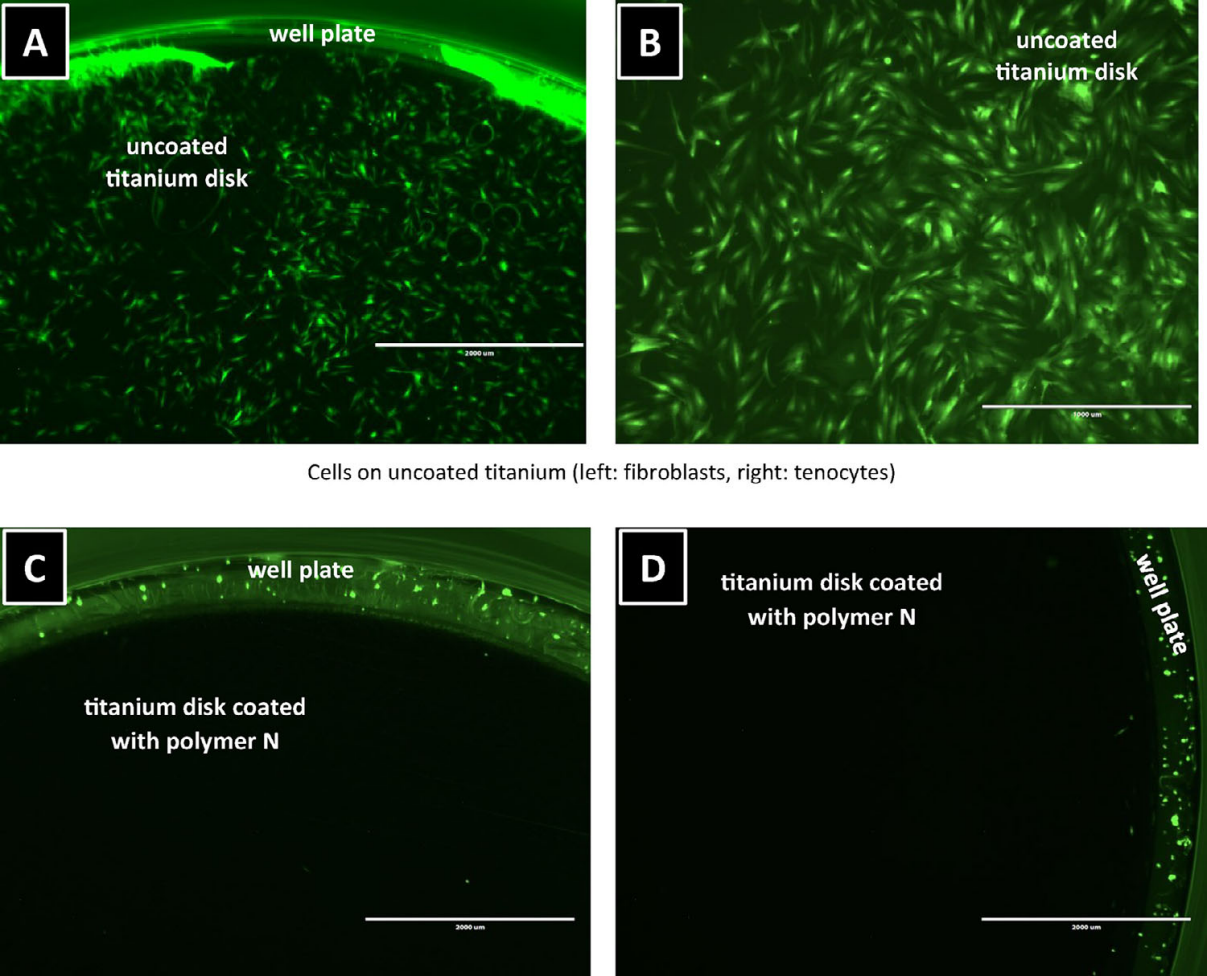
(A) Fibroblasts and (B) tenocytes seeded on uncoated titanium discs. (C) Fibroblasts and (D) tenocytes on titanium discs coated with polymer N.

**Figure** **7** demonstrates that cells adhere extensively, fully covering the surface of uncoated titanium discs (A and B). In contrast, no cell adhesion was observed on titanium surfaces coated with polymer **N**. Consistent with results from coated cell culture surfaces, the non‐adherent cells remained viable, as evidenced by their ability to adhere and proliferate when transferred to uncoated titanium discs (data not shown).

Cells seeded on unmodified cell culture plates or titanium surfaces exhibit their typical phenotype with the formation of a dense cell layer and cell‐cell contacts. Surface coating with polymer **N** abolished cell adhesion completely without being cytotoxic to the cells. This finding makes the investigated polymer coating particularly interesting for clinical applications in short‐term implants, as it offers a potential method to minimize the risks associated with explantation. To our knowledge, no clinically proven surface modifications exist that reduce tissue or tendon adhesion to titanium implants in trauma surgery. Some experimentally tested modifications based on polished titanium or in a titanium molybdenum alloy.^[^ ^42^, ^43^ ^]^ have been described in vitro and in vivo to inhibit tissue adhesion.^[^ ^44^ ^]^ Kuhn et al. described reduced fibroblast adhesion and proliferation after plasma polymerization with hexamethyldisiloxane (HMDSO).^[^ ^11^ ^]^ However, none of these modifications have made it into clinical practice.

#### 3.3.3 Bacteria Tests

The bacterium mainly responsible for infections in the field of bone osteosynthesis is Staphylococcus aureus.^[^ ^45^ ^]^ Therefore, this bacterial strain was applied to the polymer layers diluted in a medium and after 24 h the OD was measured. Moreover, the medium was seeded on agar plates and the colony forming units (CFU) was detected.

**Figure 8:**
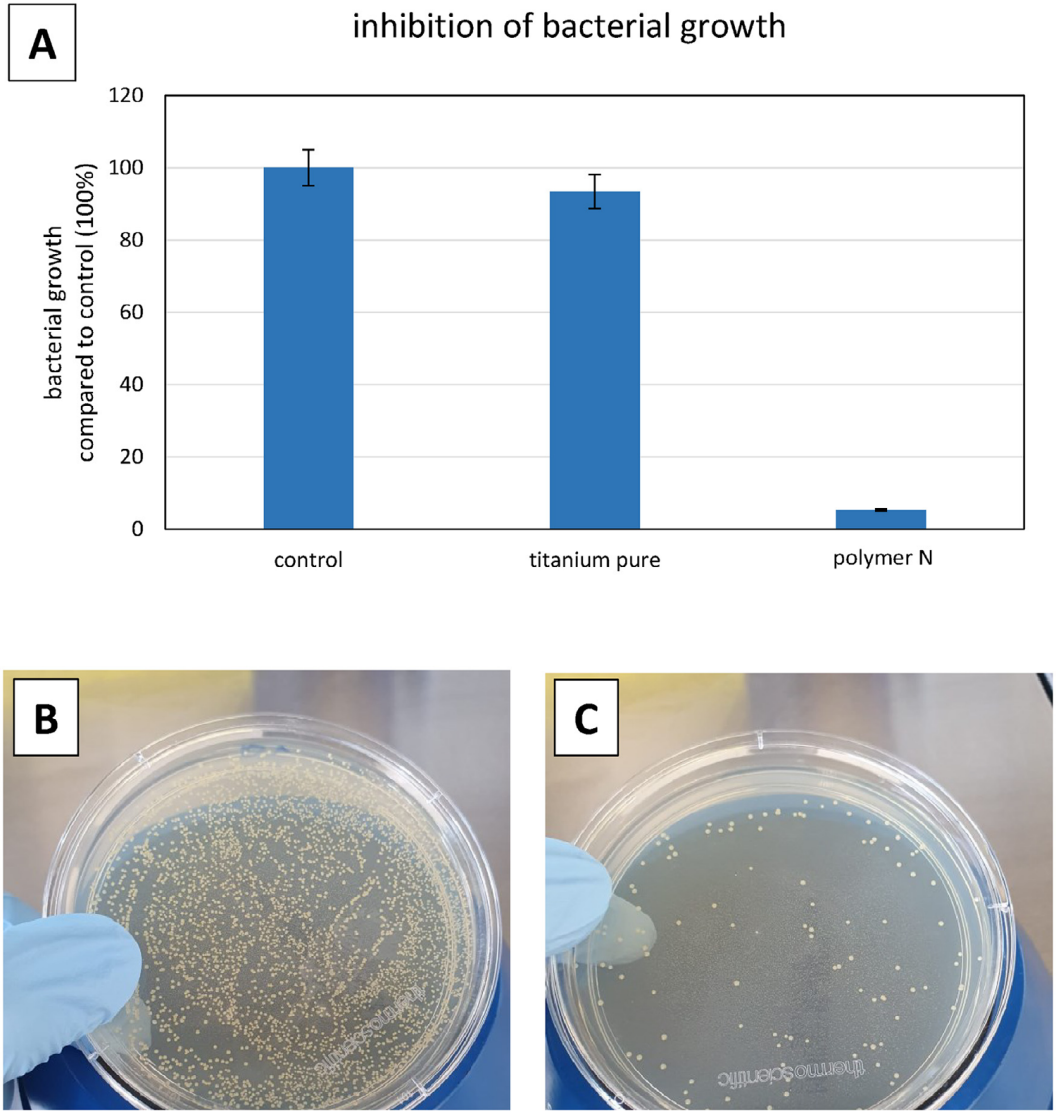
(A) Measurement of bacterial growth (OD_600_) on the coated titanium discs compared to pure LB‐medium (control 100%). CFUs 24 h after plating of the incubated medium. (B) Medium, (C) polymer N.

**Figure** **8**A shows the measurement of the OD600, calculated compared to the control (100%). Polymer **N** inhibited bacterial growth almost completely. This was confirmed by plating of the incubated medium on agar plates and analysis of the CFU. Figure 8 further exemplary shows the colonies on the control plate (Figure 8B) in comparison to polymer **N** (Figure 8C), indicating a clear anti‐microbial effect of polymer **N**.

Countless studies exist analyzing antibacterial implant modifications and some antibiotic‐releasing osteosynthesis materials are already used in clinical practice, e.g. gentamycin‐releasing bone cement. As already described in the introduction, this is associated with various possible negative side effects like promoting bacterial resistance, inducing adverse systemic effects, or failing to maintain constant antibacterial efficacy.^[^ ^13^ ^]^

Polymer **N** exhibits effective intrinsic antibacterial properties that inhibit the growth of Staphylococcus aureus without causing the typical side effects associated with antibiotic treatments. While polymers with intrinsic antimicrobial activity have been previously described,^[^ ^46^ ^]^ including their potential for immobilization on various surfaces, these materials primarily focus on preventing bacterial colonization. However, a secondary objective of many studies has been to promote tissue biocompatibility and cell adhesion. This is often achieved by combining antibacterial substrates with tissue‐adhesion‐promoting peptides, such as RGD.^[^ ^47^, ^48^ ^]^ Chitosan or streptavidin are other examples of molecules that combine intrinsic antibacterial properties with tissue‐integrating effects.^[^ ^49^, ^50^ ^]^ In contrast, our study follows a different approach. The primary aim was to develop a surface modification specifically designed for short‐term implants. This modification avoids tissue integration and adhesion while remaining non‐cytotoxic. At the same time, it effectively inhibits bacterial colonization through intrinsic antibacterial properties, thereby avoiding the adverse side effects typically associated with antibiotic treatments. This innovation is exemplified by polymer **N**.

## 4 Conclusion

This study presents a novel approach combining antiadhesive acrylamide‐based hydrogels with photocrosslinkers possessing intrinsic antibacterial properties and anchor groups specifically designed for titanium substrates.

The water‐swellable polymer networks coated on short‐term titanium implants effectively prevent cell and tissue adhesion without inducing cytotoxicity. Based on the non‐adhesiveness, such polymer layers are expected to circumvent injuries during implant removal caused by tight implant tissue integration. Intrinsic antibacterial effects have been integrated into the molecular framework of these coatings via the specific structure of the cationic crosslinker BPQAAm and have been demonstrated to successfully inhibit bacterial colonization. This avoids the use of additional antibiotics or bactericidal agents that tend to leach from the coating in a rather uncontrolled manner. Based on these findings this innovative coating design demonstrates significant potential to advance short‐term titanium implant technology, addressing key challenges related to tissue adhesion and infection risks.

Future investigations should focus on further validating the performance of the coatings in appropriate in vivo models. Successful translation of this coating technology into clinical applications could lead to reduced complications associated with the removal of short‐term trauma implants, eventually improving therapeutic results and patient well‐being.

## Conflict of Interest

Patent: WO2024094645A1‐“Nonstick polymer coatings for short‐term implants”, Inventors: Niklas Jung, Ulrich Jonas, Fiona Wiesner (née Diehl), Clinton Richard Viktor Thiagarajan, Ulrike Ritz, Karl Michael Georg Schönewald, Kira Vogel

## Supporting information



Supporting Information

## Data Availability

The data that support the findings of this study are available in the supplementary material of this article.
