# Supplementary material for: Antiadhesive and Antibacterial Coatings for Short‐Term Titanium Implants
Source: Macromol Rapid Commun. 2025 Mar 8;46(12):2400989. doi: 10.1002/marc.202400989 (PMC12183145; doi:10.1002/marc.202400989)
Supplement: Supplementary file 1 — Supporting Information [file MARC-46-2400989-s001.pdf]

Macromolecular Rapid Communications

Supporting Information

for *Macromol. Rapid Commun.*, DOI 10.1002/marc.202400989

Antiadhesive and Antibacterial Coatings for Short-Term Titanium Implants

Fiona Wiesner, Karl G. M. Schönewald, Kira Vogel, Niklas Jung, Barbara Schwierz, Maren Kipping, Aliia Ibragimova, Joshua Schumacher, Christian Pritzel, Clinton R. V. Thiagarajan, Ulrike Ritz and Ulrich Jonas**

Supporting Information

Antiadhesive and Antibacterial Coatings for Titanium Implants

Fiona Wiesner,[1] Karl G. M. Schönewald,[2] Kira Vogel,[2] Niklas Jung,[1] Barbara Schwierz,[2] Maren Kipping,[1] Aliia Ibragimova,[1] Joshua Schumacher,[3] Christian Pritzel,[4] Clinton R. V. Thiagarajan,[1] Ulrike Ritz,*[2] and Ulrich Jonas*[1]

[1]University of Siegen, Department of Biology and Chemistry, Macromolecular Chemistry, Adolf-Reichwein-Straße 2, 57076 Siegen, Germany – Email: jonas@chemie.uni-siegen.de

[2]University Medical Center Mainz, Technology Transfer, Langenbeckstraße 1, 55131 Mainz, Germany

[3]University of Siegen, Department of Biology and Chemistry, Physical Chemistry I, Adolf-Reichwein-Straße 2, 57076 Siegen, Germany

[4]University Medical Center Mainz, Department of Orthopedics and Trauma Surgery, Langenbeckstraße 1, 55131 Mainz, Germany – Email: ritz@uni-mainz.de

[5]University of Siegen, Department of Biology and Chemistry, Chemistry of Building Materials, Paul-Bonatz-Straße 9-11, 57076 Siegen, Germany

*contributed equally, corresponding authors

S1. Polymer Synthesis

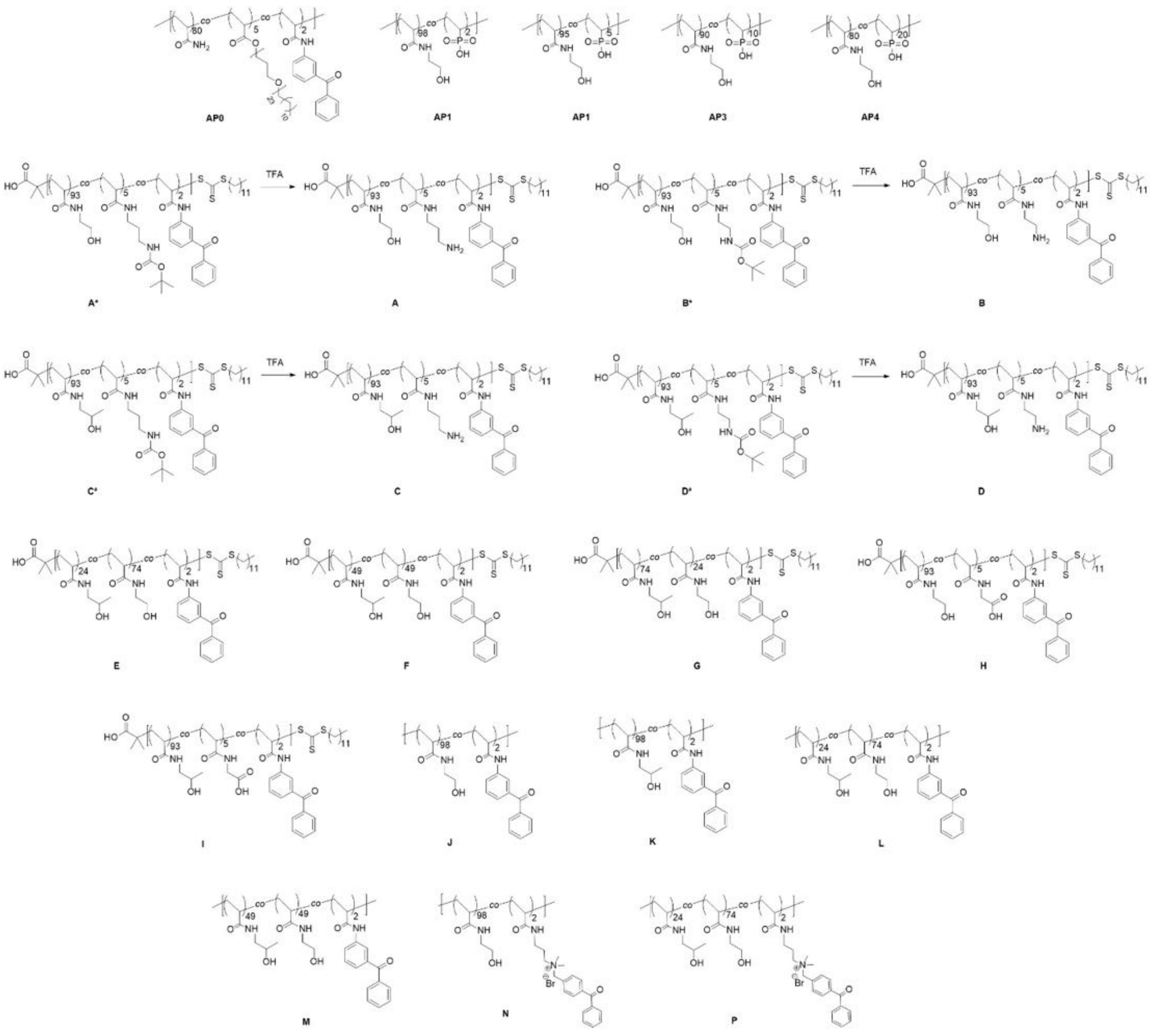

Figure S1. Overview about all synthesized polymers including their abbreviations.

Table S1. Educts, yields, and GPC results for the copolymerization of VPA and HEAm with different built-in ratios of VPA (*c*(VPA)). Synthetic details for the adhesion promoter polymer AD0 can be found in literature. [1]

**#**	***c*(VPA)**	**VPA[a,b]**		**HEAm[a,b]**		**AIBN[a]**		**Yield**		**GPC**	
	%	mg	µmol	mg	µmol	mg	µmol	mg	%	$\overline{M}_n$ / kg mol^{-1}	*Đ*
**AP1**	2	3.84	35.5	200	1.74	1.46	8.88	170	83	30	1.6
**AP2**	5	9.87	91.4	200	1.74	1.50	9.16	180	85	37	2.1
**AP3**	10	20.8	193	200	1.74	1.59	9.67	189	85	49	2.1
**AP4**	20	46.9	434	200	1.74	1.79	10.9	204	82	31	3.0

[a] taken from a stock solution in MeOH (*β* = 100 mg/ml) in case of #**1** to #**4**.

Table S2. Educts, yields, and GPC results for the RAFT copolymerization of the polymers **A** - **I** with different comonomers.

#	**Monomers** Name / mg(mmol)		**BPAm** / mg (µmol)	**DMP** / mg (µmol)	**AIBN** / mg (µmol)	**Yield** %	**GPC** $\overline{M_n}$ / kg mol^{-1}	*Đ*
**A***	HEAm 362 (3.14)	APAm-Boc 38.0 (0.17)	16.61 (66.2)	6.02 (17.0)	2.80 (17.4)	92	35	1.3
**B***	HEAm 364 (3.16)	AEAm-Boc 36.0 (0.17)	16.72 (66.6)	6.06 (17.0)	2.80 (17.4)	98	33	1.2
**C***	HPAm 366 (2.83)	APAm-Boc 34.0 (0.15)	15.07 (60.0)	5.44 (15.0)	2.40 (15.0)	76	15	1.1
**D***	HPAm 367 (2.84)	AEAm-Boc 32.0 (.015)	15.07 (60.0)	5.46 (15.0)	2.40 (15.0)	78	11	1.2
**E**	HEAm 289 (2.51)	HPAm 110 (0.85)	17.2 (67.4)	6.12 (16.8)	2.80 (17.4)	83	35	1.2
**F**	HEAm 198 (1.72)	HPAm 212 (1.73)	16.73 (65.6)	6.28 (17.3)	2.80 (17.4)	73	39	1.2
**G**	HPAm 308 (2.38)	HEAm 97 (0.84)	16.23 (63.6)	5.86 (16.1)	2.80 (17.4)	66	36	1.4
**H**	HEAm 377 (3.27)	AaAm 22.0 (0.17)	17.33 (69.0)	6.28 (17.3)	2.80 (17.4)	67	32	1.3
**I**	HPAm 380 (2.94)	AaAm 20.0 (0.15)	15.58 (61.8)	5.63 (15.5)	2.50 (15.5)	66	34	1.4

Table S3. GPC data before and after deprotection of the RAFT copolymers **A** - **D**.

#	Monomers	GPC (before deprotection) $\overline{M_n}$ / kDa	Đ	GPC (after deprotection) $\overline{M_n}$ / kDa	Đ	Yield / mg	/ %
**A**	HEAm - APAm	34.7	1.3	34.6	1.3	92	92
**B**	HEAm - AEAm	38.0	1.2	33.3	1.2	98	98
**C**	HPAm - APAm	39.3	1.2	14.5	1.2	38	76
**D**	HPAm - AEAm	32.0	1.3	10.9	1.2	39	78

Table S4. Educts, yields, and GPC results for the FRP of the polymers **J** - **P** with different comonomers.

#	HEAm / mg (mmol)	HPAm / mg (mmol)	Crosslinker Name / mg (µmol)	AIBN / mg (µmol)	Yield %	GPC $\overline{M_n}$ / kDa	Đ
**J**	500 (4.34)	-	BPAm 22.3 (88.6)	3.64 (22.2)	79	52	3.2
**K**	-	400 (3.10)	BPAm 15.58 (62.0)	2.50 (15.5)	79	60	2.3
**L**	500 (4.34)	182 (1.41)	BPAm 29.5 (117)	4.82 (29.3)	82	69	3.8
**M**	212 (1.73)	198 (1.72)	BPAm 16.73 (65.6)	2.80 (17.4)	75	63	2.5
**N**	500 (4.34)	-	BPQAAm 38.2 (88.6)	3.64 (22.2)	83	40	3.8
**P**	289 (2.51)	110 (0.85)	BPQAAm 29.1 (67.4)	2.82 (17.2)	91	42	2.2

S2. Polymer Analysis

S2.1 NMR Spectroscopy

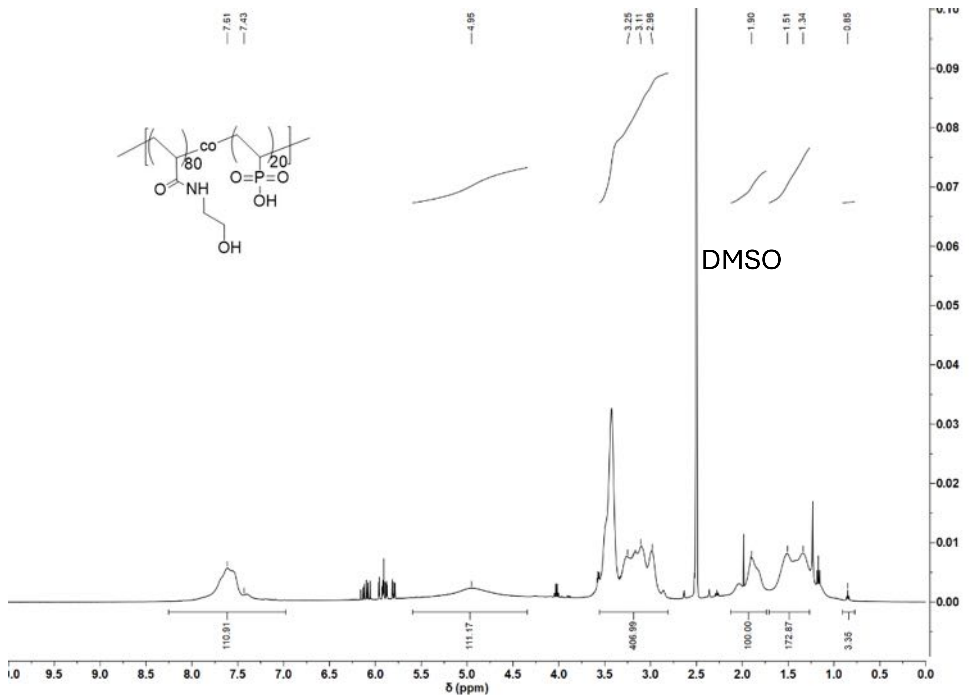

Figure S2. ^{1}H NMR spectrum of **AP4** measured in DMSO-d6.

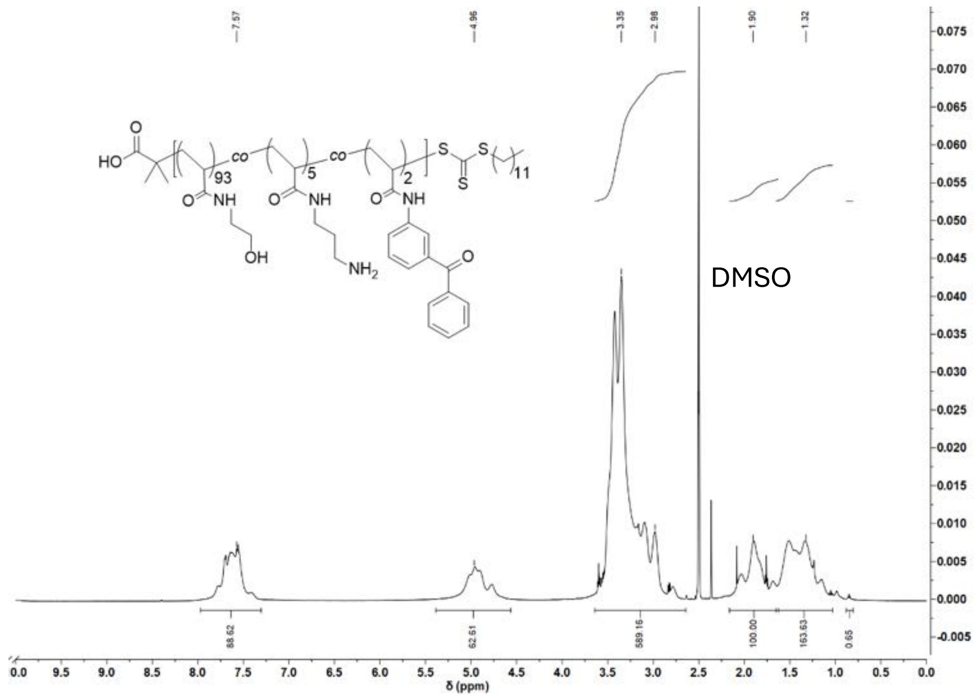

Figure S3. ^{1}H NMR spectrum of polymer **A**, measured in DMSO-d6.

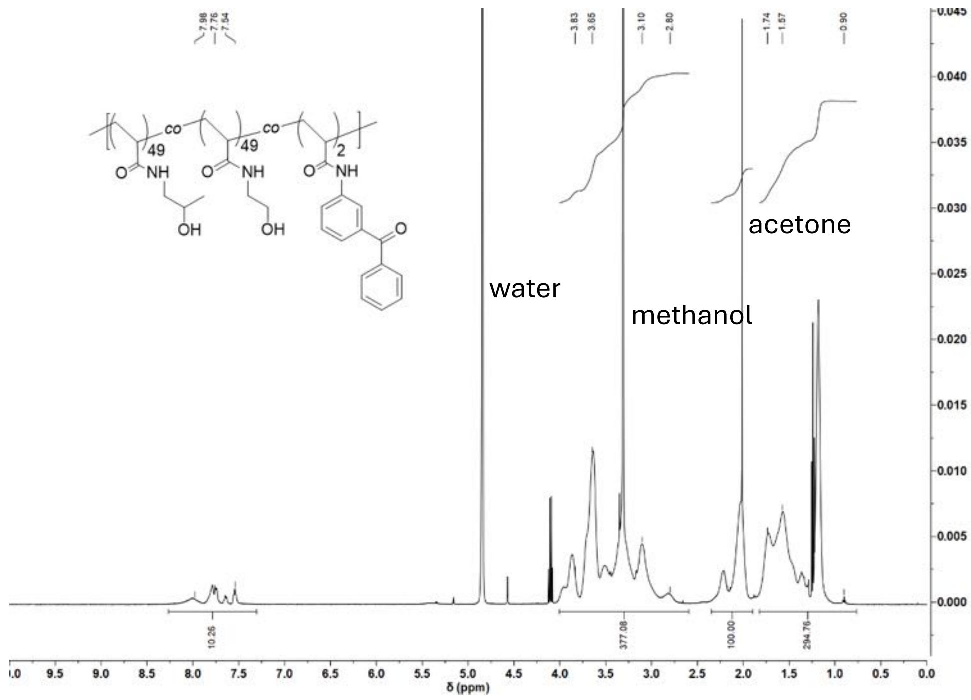

Figure S4. ^{1}H NMR spectrum of polymer **M**, measured in methanol-d3.

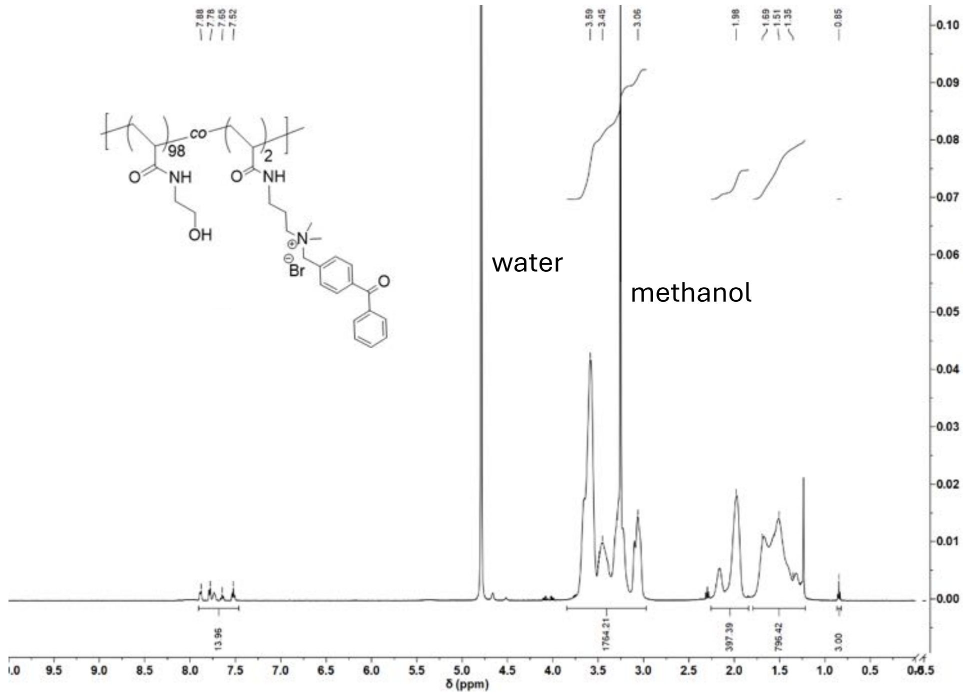

Figure S5. ^{1}H NMR spectrum of polymer **N**, measured in methanol-d3.

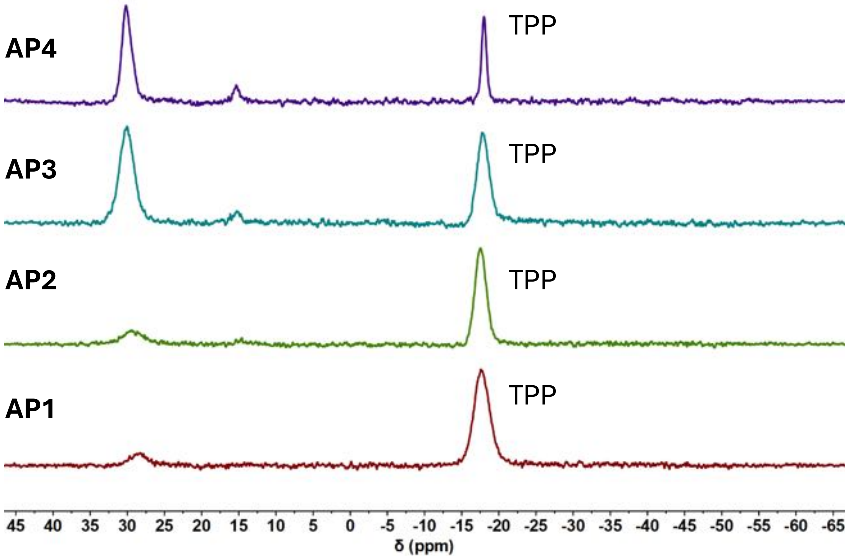

Figure S6. ^{31}P NMR measurement of the anchor polymers **AP1** – **AP4** with triphenyl phosphate (TPP) as standard.

S3. Polymer Network Analysis

S3.1 Contact Angle Measurements

Static contact angles measurements

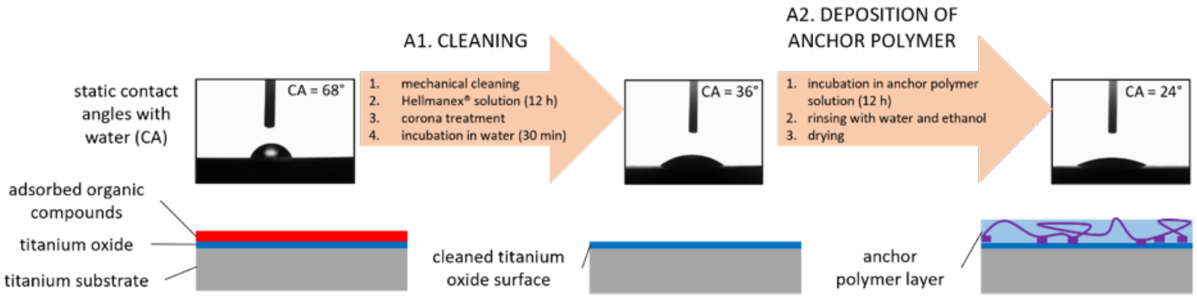

Figure S7. Procedure for performing the static contact angle measurements.

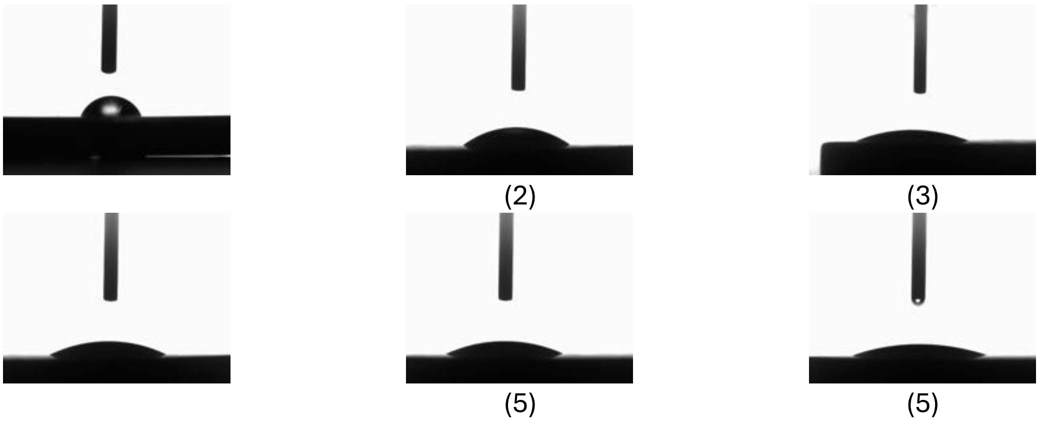

Figure S8. Static contact angles with water on (1) Ti as-received (CA = 68°), (2) cleaned titanium (CA = 36°), (3) Ti with **AP1** (CA = 22°), (4) Ti with **AP2** (CA = 25°), (5) Ti with **AP3** (CA = 24°), and (6) Ti with **AP4** (CA = 18°).

Dynamic contact angle measurements

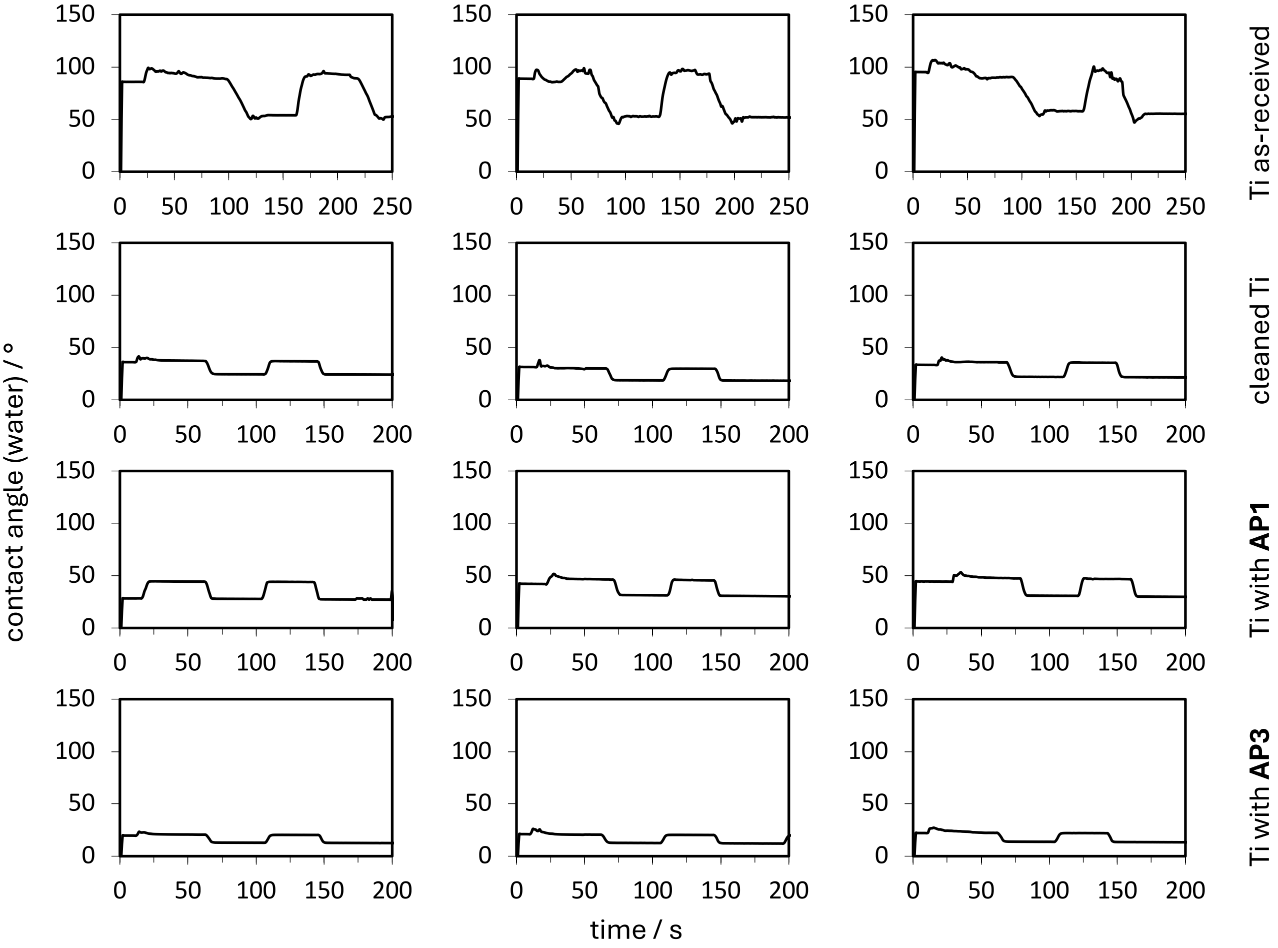

Figure S9. Graphs of the dynamic contact angle measurements with water.

S2.2 Environmental Scanning Electron Microscopy

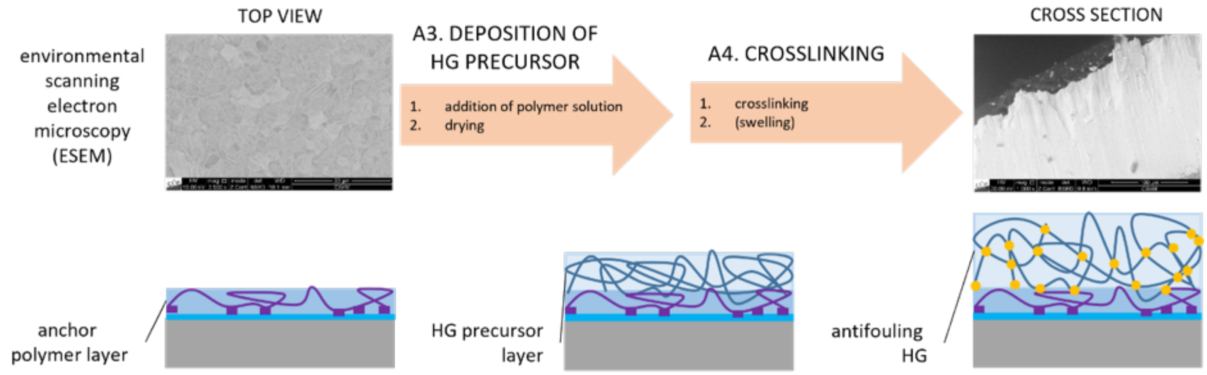

Figure S10. Workflow for the ESEM-EDX measurements.

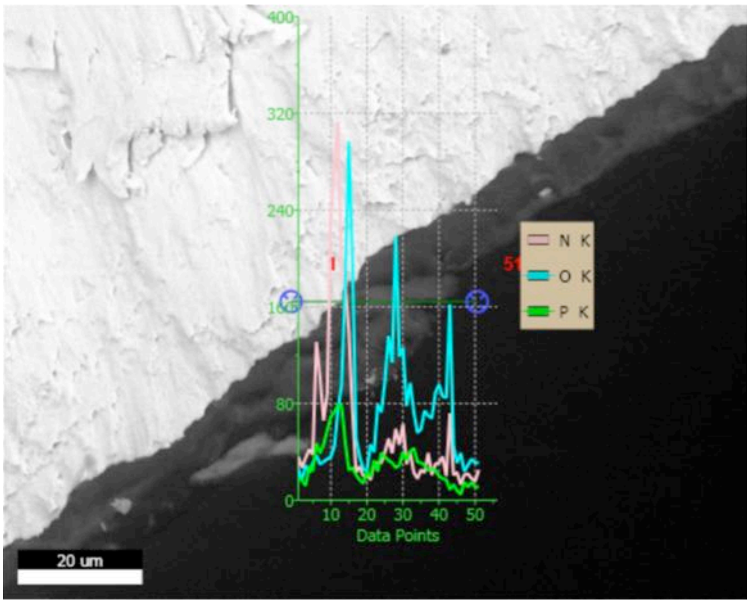

Figure S11. Results of the EDX line scan of the cross-section (raw data).

S2.3 Atomic Force Microscopy

Preparation of the cantilever

The following procedure was adapted from Norman et al. [2] To place the adhesive onto the end of the cantilever, the tipless cantilever was mounted into the AFM holder. The adhesive was dropped onto a microscopy slide, which was placed in the sample holder of the AFM. The optical camera system was used to bring the cantilever into contact with the adhesive, allowing the distance between the two to be controlled visually. Contact-mode force curves were acquired in order to carefully dip the end of the cantilever into the adhesive, allowing an intimate contact with a dwelling time of 10 seconds and a trigger of 0.1 V. Afterwards, a microscopy slide coated with silica beads was placed in the sample holder of the AFM. The cantilever was carefully lowered above a bead visible in the optical camera system and then force curves with a deflection volt trigger of 1 V were recorded until the cantilever touched the bead and the adhesive was allowed to get into contact with the bead for a dwelling time of 10 seconds. In order to fix the attached

bead to the cantilever, the modified cantilever was exposed to UV irradiation for 15 minutes at a wavelength of 365 nm. After conducting the force measurements, the cantilever was sputtered with gold for SEM imaging.

Quality assessment and calibration of the modified cantilever

The successful modification of the cantilever was checked by scanning electron microscopy and by AFM. By measuring an AFM height image over a calibration grid made of sharp and upwards-facing cantilever-tips, the three-dimensional structure of the attached bead could be visualized and confirmed a highly homogeneous surface of the bead as can be seen in Figure S12.

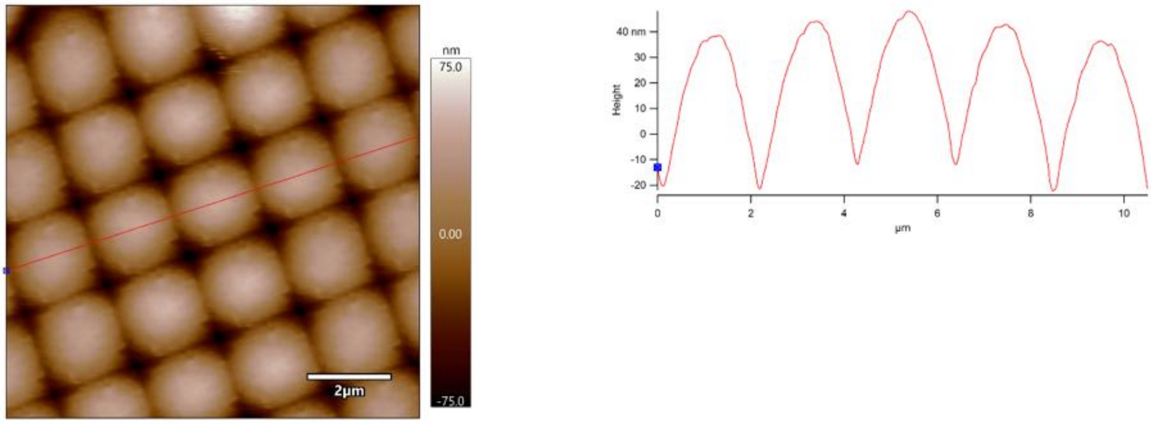

Figure S12. AFM height image of the calibration grid exposing upwards-facing AFM-tips (left) and cross-section analysis (right).

Additionally, the cantilever was imaged by electron microscopy as can be seen in Figure S13.

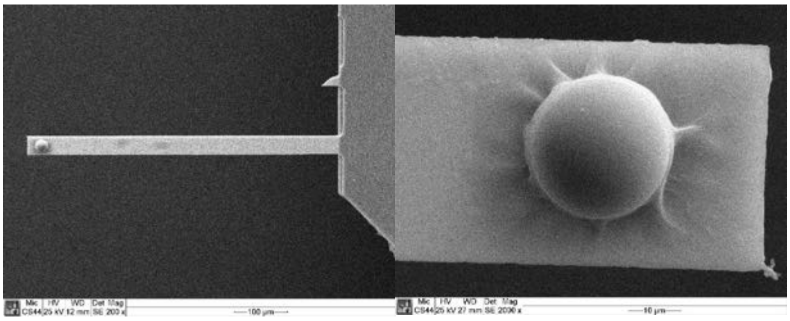

Figure S13. SEM secondary electron image of the top view of the modified cantilever (left) and a higher magnification of the bead with the sample holder tilted by 45° (right).

In the SEM images, the highly homogeneous glass bead can be seen without any impurities in the contact region. The bead-modified cantilever was then mounted to the AFM and force curves were recorded in order to determine the elastic modulus of the hydrogel-coated substrate. In order to determine said value, the spring constant was determined based on the thermal calibration method. For that, the virtual deflection was measured by measuring a force curve over the maximum distance possible in air without having a trigger. The change in the deflection of the cantilever during the z-movement was fitted and automatically subtracted from the subsequent measurements. Afterwards, the mounted cantilever was pressed 200 times on a glass slide which was previously cleaned using an acetone-saturated wipe. By plotting the measured deflection

against the z-distance and fitting the contact part of this fit, the inverse optical lever sensitivity was determined. For that, the determined slopes for all force curves were plotted in a histogram and the mean value was calculated based on a Gaussian fit. In order to determine the cantilevers spring constant, the temperature-induced oscillations of the cantilever were recorded and the resonance frequency was fitted according to the equipartition theorem.[1] Knowing the inverse optical lever sensitivity, the cantilevers spring constant could be determined and was found to be 250 ± 30 pN nm^{-1}. This process was repeated three times and the mean value was calculated. After determining the spring constant, the InvOLS value could be determined prior to new experiments based on the thermal spectrum of the cantilever as described before, ranging from 400 to 550 nm/V depending on the position of the laser on the cantilever.

Force probing on the hydrogel in the dry state revealed a relatively stiff surface which cannot be indented by the very soft cantilever as can be seen in Figure S14.

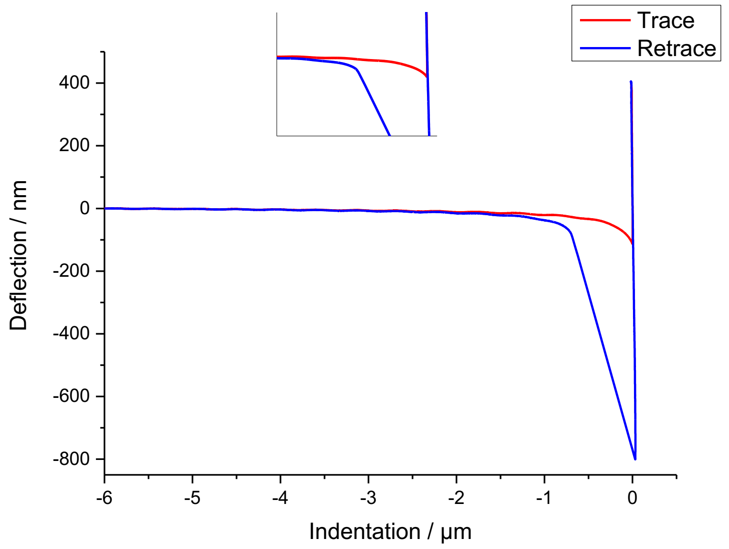

Figure S14. Deflection-indentation plot of the dry network from the hydrogel precursor polymer, measured in air.

Upon approaching the surface, long-range attractive forces lead to a bending of the cantilever towards the surface. As soon as the cantilever touches the surface, no indentation is observed, but only bending of the cantilever. Fitting of the force curve according to the Hertzian model yields apparent moduli exceeding 200 MPa. For this reason, the cantilever with a spring constant of 250 pN/nm is not suited for indentation measurements of the dry polymer network.

Force measurements in water on the swollen hydrogel coating yielded the force curves shown in Figure S15, where no adhesion is present and indentation of the hydrogel layer can be observed.

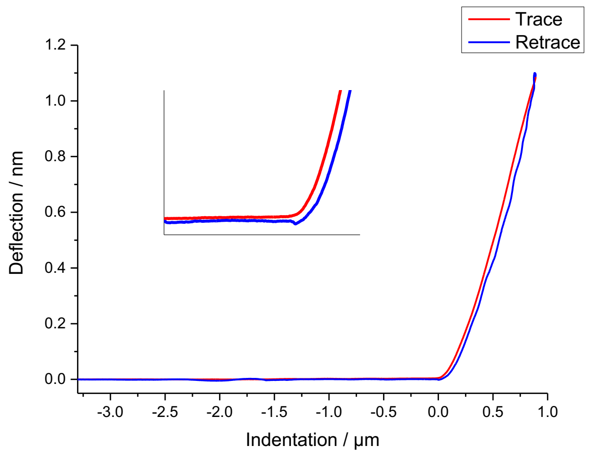

Figure S15. Force-indentation plot of the swollen hydrogel coating measured in water.

After measuring the force maps, by fitting the Hertzian model to the approach curves as can be seen in Figure S16 the Young´s modulus was determined. Inhomogeneous force curves were neglected.

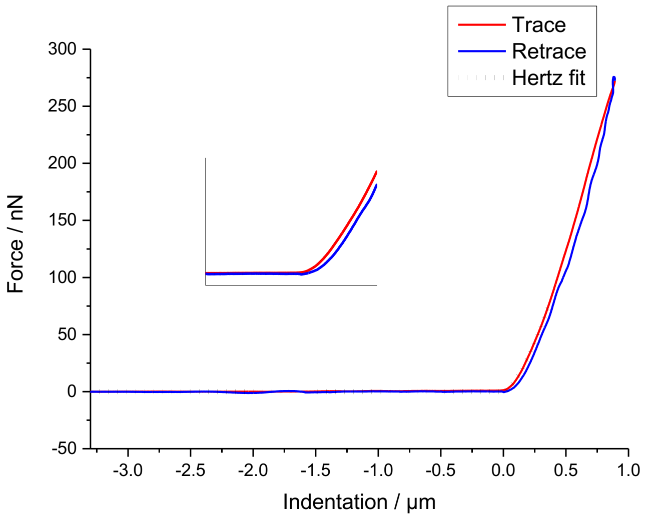

Figure S16. Force-indentation plot of the swollen hydrogel measured in water. The dotted black line corresponds to the Hertzian fit.

The measured Young´s moduli yield a distribution as can be seen in Figure S17. Eighteen force maps were excluded due to inhomogeneities during the measurement of those curves measured. A Gaussian fit has been fitted to the data to determine the mean value and the full width half maximum is taken to describe the distribution of the Young´s moduli.

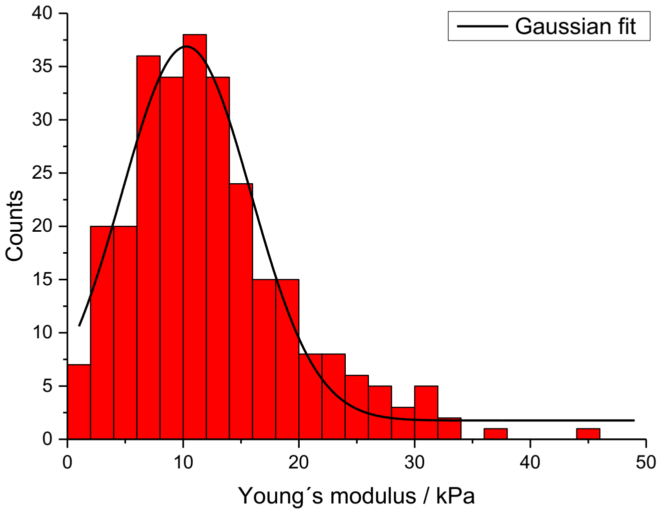

Figure S17. Histogram of the Young´s moduli determined from three separate force maps.

S2.4 Coverage of the Titanium Specimens

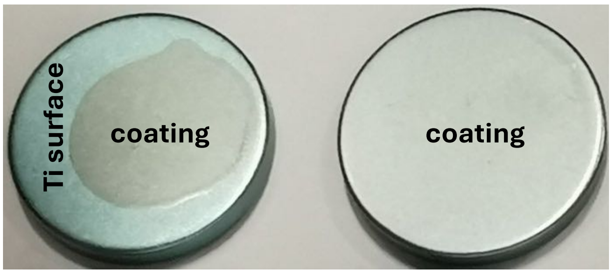

Figure S18. Exemplary photograph of two polymer network-coated titanium disks with the specimen on the left showing a partially (drop-) coated sample and the specimen on the right an completely covered surface.

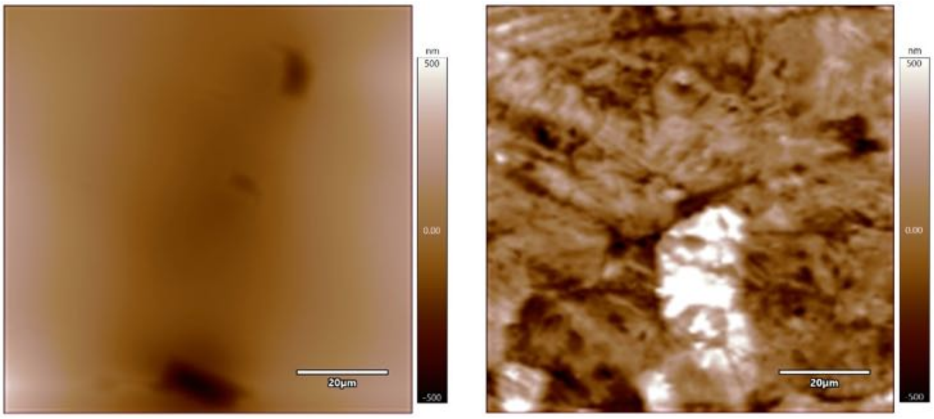

Figure S19. AFM surface scans in tapping mode of (left) the dry polymer coating (ambient conditions) and (right) the untreated titanium surface.

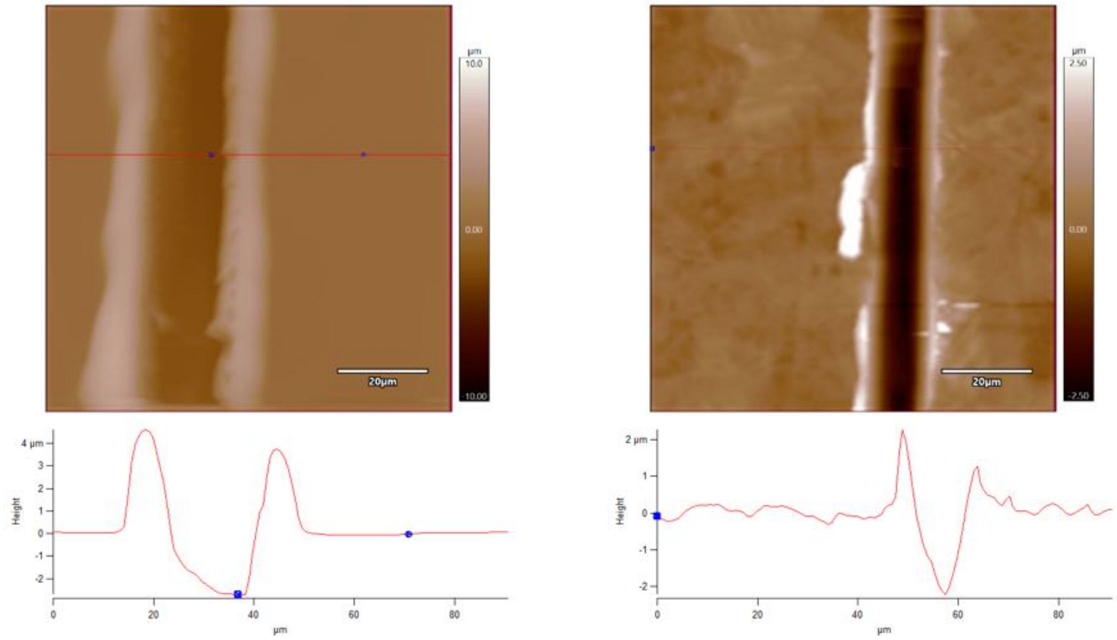

Figure S20. AFM surface scans in tapping mode of a sample scratched with a scalpel: (left) sample previously coated with polymer (ambient conditions) and (right) untreated titanium surface. The bottom pictures depict their corresponding height profiles (red line in the surface map visualizes the height profile line scan).

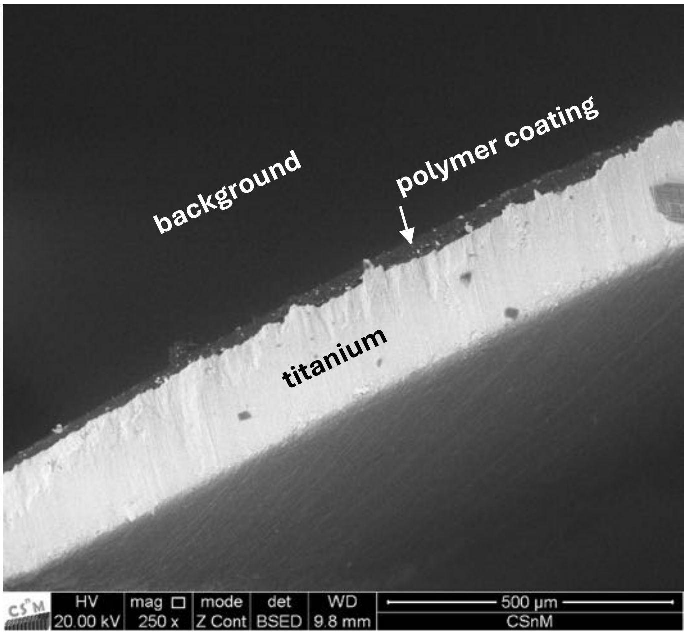

Figure S21. ESEM picture of the cross section of a coated titanium substrate, showing even surface coverage.

Literature

1 Freese, S.; Diraoui, S.; Mateescu, A.; Frank, P.; Theodorakopoulos, C.; Jonas, U. Polyolefin-Supported Hydrogels for Selective Cleaning Treatments of Paintings. *Gels* **2020**, *6*, doi:10.3390/gels6010001.

2 Cook, S. M.; Lang, K. M.; Chynoweth, K. M.; Wigton, M.; Simmonds, R. W.; Schäffer, T. E. Practical Implementation of Dynamic Methods for Measuring Atomic Force Microscope Cantilever Spring Constants. Nanotechnology **2006**, *17* (9), 2135–2145. https://doi.org/10.1088/0957-4484/17/9/010.
